# Genome-Wide Evolutionary Characterization and Expression Analyses of WRKY Family Genes in *Brachypodium distachyon*

**DOI:** 10.1093/dnares/dst060

**Published:** 2014-01-21

**Authors:** Feng Wen, Hong Zhu, Peng Li, Min Jiang, Wenqing Mao, Chermaine Ong, Zhaoqing Chu

**Affiliations:** Shanghai Chenshan Plant Science Research Center, Shanghai Institutes for Biological Sciences, Chinese Academy of Sciences (CAS), Shanghai Chenshan Botanic Garden, 3888 Chenhua Road, Songjiang, Shanghai 201602, China

**Keywords:** WRKY, *Brachypodium distachyon*, evolution, abiotic stresses, biotic stresses

## Abstract

Members of plant *WRKY* gene family are ancient transcription factors that function in plant growth and development and respond to biotic and abiotic stresses. In our present study, we have investigated *WRKY* family genes in *Brachypodium distachyon*, a new model plant of family Poaceae. We identified a total of 86 *WRKY* genes from *B. distachyon* and explored their chromosomal distribution and evolution, domain alignment, promoter *cis*-elements, and expression profiles*.* Combining the analysis of phylogenetic tree of *BdWRKY* genes and the result of expression profiling, results showed that most of clustered gene pairs had higher similarities in the WRKY domain, suggesting that they might be functionally redundant. Neighbour-joining analysis of 301 WRKY domains from *Oryza sativa*, *Arabidopsis thaliana*, and *B. distachyon* suggested that BdWRKY domains are evolutionarily more closely related to *O. sativa* WRKY domains than those of *A. thaliana*. Moreover, tissue-specific expression profile of *BdWRKY* genes and their responses to phytohormones and several biotic or abiotic stresses were analysed by quantitative real-time PCR. The results showed that the expression of BdWRKY genes was rapidly regulated by stresses and phytohormones, and there was a strong correlation between promoter *cis*-elements and the phytohormones-induced *BdWRKY* gene expression.

## 1. Introduction

Grasses (Poaceae), including rice, wheat, and sorghum, are the most important plant species on the earth, and are a major source of nutrition and sustainable energy.^1^ To study Poaceae genome will help scientists better understand the mechanisms of how genes control physiological events in Poaceae, and help discover and make use of functional genes from the large amount of Poaceae plants, especially from those able to survive under extreme conditions. Recently, *Brachypodium distachyon* has been used as a new model organism for Poaceae grass, as it is much more closely related to several economically important Poaceae species such as rice, sorghum, wheat, and turf grasses.

The *WRKY* family genes are plant transcription activators in various physiological processes; they were regarded as the first isolated regulatory genes from plants.^2,3^ WRKY transcription factors (TFs) are conserved in evolutional history throughout the plant kingdom. Members of this family contain at least one conserved DNA-binding domain with a highly conserved WRKYGQK heptapeptide sequence, followed by a C_2_H_2_- or C_2_HC-type of zinc finger motifs. These conserved sequences have been designated as the WRKY domains, and function in W-box DNA motif (C/T)TGAC(C/T)-binding activation.^4^ In *Arabidopsis thaliana*, a total of 72–74 members of the WRKY TFs can be divided into three major groups with several subgroups, based on their sequences in the WRKY domain and their relationships in the phylogenetic clades.^4,5^ The Group I WRKY TFs contain two WRKY domains, one at the C- and the other at the N-terminal of the protein. These two WRKY domains seem to be functionally redundant.^6^ Peptide sequences outside the C-terminal WRKY domain contribute significantly to the overall strength of DNA binding; the N-terminal WRKY domain might participate in the binding process by increasing the affinity or specificity to their targets.^7–9^ In contrast, most Group II and Group III WRKY TFs only contain a single WRKY domain; this domain is more similar in sequence to the C-terminal than to the N-terminal WRKY domain of Group I proteins, suggesting that the C-terminal WRKY domain in Group I WRKY TFs and single WRKY domains in Group II and Group III WRKY TFs are functionally equivalent and constitute the major DNA-binding domain.^4^ The differences between Groups II and III are in their C-terminal zinc domain.

Previous studies have demonstrated that WRKY TFs play essential roles in various physiological processes, including senescence, root development, sugar signalling, and germination.^3,10^ Furthermore, WRKY TFs have been shown to be involved in responses to various biotic stresses caused by viruses,^11^ bacterial pathogens,^12,13^ fungi,^14^ abiotic stresses,^3,15,16^ and some signalling substances such as salicylic acid (SA)/benzothiadiazole,^17–19^ jasmonic acid (JA),^18–20^ gibberellin,^21^ and abscisic acid (ABA).^22,23^ In *Arabidopsis*, the majority of the 74 WRKY genes are transcriptionally inducible upon pathogen infection and other defence-related stimuli.^24^ For example, it has been proven that AtWRKY25 functioned as a negative regulator of SA-mediated defence responses to *Pseudomonas syringae*.^13^ In *Boea hygrometrica* leaves, BhWRKY1 is proven to be a regulator in an ABA-dependent signal pathway to regulate BhGolS1 expression.^23^ Using northern blotting analysis, Qiu *et al.*^15^ revealed in rice that 10 of 13 *OsWRKY* genes were differentially regulated in response to abiotic stress factors NaCl, polyethylene glycol (PEG), cold, and heat. Under a salinity stress, a microarray analysis using 70-mer oligonucleotide probes representing 23 686 genes revealed that 18 *AtWRKY* genes were induced in *A. thaliana* root treated with 150 mM NaCl.^16^ Furthermore, numerous studies have shown that many *WRKY* genes were responsive to drought, heat, cold, and so on. On the other hand, a single *WRKY* gene often showed transcription activity in response to several stress factors, indicating that it has different regulatory function in diverse stress responses. For example, the expression of *AtWRKY25* and *AtWRKY33* were responsive to both heat and salt stress.^25,26^ Thus, a genome-wide analysis of *B. distachyon WRKY* genes should help to reveal the underlying complex molecular mechanisms of WRKY proteins in response to various stresses.

In our study, 86 *WRKY* genes were identified from the *B. distachyon* Bd21 genome and classified according to their homology with known *WRKY* genes in *Oryza sativa*. We investigated the evolutionary relationship of *B. distachyon* WRKY TFs with their counterparts from monocot *O. sativa* and dicot *A. thaliana*. Subsequently, we used quantitative real-time PCR (qRT-PCR) to examine their transcript profiles in different tissues and in response to several biotic or abiotic stresses and phytohormone treatments. Since *BdWRKYs* showed various expression patterns and expression levels under a series of abiotic stresses and phytohormone treatments, we checked if there are correlations between the differences in the WRKY domain and their spatial and temporal expression patterns in response to stress treatments. We have also done detailed correlation analyses between promoter *cis*-elements and the genes expression pattern. Our study provided genome-wide evolutionary characterization and expression analysis of *WRKY* genes in *B. distachyon*, an important step for further investigation into the functions of these genes.

## 2. Materials and methods

### 2.1. Sequence retrieval

We performed a BLAST search among sequenced genomes of land plants in plantTFDB,^27^ GramineaeTFDB,^28^ Superfamily, and Phytozome (http://www.Phytozome.net) using well-known plant WRKY proteins as queries. The database of UniProt (http://www.uniprot.org/blast/) and GeneBank (http://www.ncbi.nlm.nih.gov/) were used for searching the WRKY proteins in red and green algae. To verify the reliability of our results, all putative non-redundant sequences were assessed with UniProt and SMART (http://smart.embl-heidelberg.de/) analyses, respectively.

### 2.2. Identification of WRKY protein in *B. distachyon*

To identify *B. distachyon* genes encoding WRKY proteins with at least one possible WRKY domain, we performed a GeneBank BLASTP search, UniProt, and *B. distachyon* genome Database (http://www.brachypodium.org/), using the amino acid sequences of the WRKY domain. The Brachy WRKY Database (http://www.igece.org/WRKY/BrachyWRKY/BrachyWRKYIndex.html) was used as a referral for verifying the reliability of our results.^29^ We also obtained information of the chromosome locations of each gene from the results of BLASTP at the *B. distachyon* genome Database. A total of 86 *BdWRKY* genes were found in *B. distachyon* (Supplementary Table S1). Furthermore, to avoid confusion, we used the same numbering system as Tripathi *et al.*^29^

### 2.3. Sequence analysis

To analyse the sequence of the 86 typical identified *B. distachyon* WRKY proteins, we performed multiple alignment analyses of the WRKY domains sequence by ClustalW (www.ebi.ac.uk/clustalw/).^30^

### 2.4. Phylogenetic analysis

A neighbour-joining (NJ) tree was constructed using the MEGA version 5 software,^31^ based on the alignment of WRKY domains in *O. sativa*, *A. thaliana*, and *B. distachyon*. To determine the statistical reliability, we conducted bootstrap analysis with the following parameters: p-distance and pairwise deletion. Bootstrap analysis was performed with 1000 replicates.

### 2.5. Protein motifs and structure analysis

Analysis for conserved motifs in the WRKY proteins was carried out using MEME (http://meme.sdsc.edu/meme/cgi-bin/meme.cgi).^32^ The settings were: any number of repetitions of a single motif, the minimum width of a motif with six amino acids, the maximum width of a motif with 80 amino acids, and the maximum number of motifs up to 15 amino acids. Subsequently, the MAST program was used to search detected motifs in protein databases.^33^ The details of sequence logo of motifs were shown in Supplementary Fig. S1.

### 2.6. Cluster analysis of expression data

The 2-week-old seedlings (Bd21) were used for harvesting leaf, stem, and root samples. For phytohormone analysis, 2-week-old seedlings were treated in MS liquid medium containing 100 µM methyl jasmonate (MeJA), 100 µM ABA, 1 mM SA, and 20 µM 6-Benzylaminopurine (6-BA) for 3 h, respectively. For abiotic stress treatment, 2-week-old seedlings were treated in MS liquid medium containing 20% PEG, 200 mM NaCl, and 10 mM H_2_O_2_ for 3 h, respectively. Cold and heat treatments were achieved by placing 2-week-old seedlings in MS liquid medium at 4 or 45°C for 3 h, respectively. For phytopathogen treatment, 2-week-old seedlings were sprayed with *Fusarium graminearum* (F0968) and two strains of *Magnaporthe grisea* (Guy11, avirulent ACE1 genotype; PH14, virulent ACE1 genotype) for 4 or 12 h. The *BdWRKY* array constituted of 86 primer sets representing all members of the *B. distachyon WRKY* gene family. The primer sets are listed in Supplementary Table S2. The expression of the 86 *BdWRKY* genes was assessed upon the qPCR result analysis. Each experiment was repeated three separate times. The expression profile was calculated from the –ΔΔCT value [−ΔΔCT = (CTcontrol.gene − CTcontrol.actin) - (CTtreat.gene − CTtreat.actin)], and obtained by the PermutMatrixEN vesion 1.9.3 software, and shown by a green-red gradient. The data were statistically analysed using an OriginPro 7.5 software. The up-regulated genes were defined as a fold change greater than 1.5 with a *P*-value of <0.05, and with a fold change of ≤0.66 was used to define down-regulated genes when the *P-*value of <0.05.

### 2.7. Promoter analysis

The 1500 bp promoter sequences of *BdWRKY* genes were obtained from the *B. distachyon* genome Database. PLANT CARE (http://bioinformatics.psb.ugent.be/webto​ols/plantcare/html/) was used to determine the *cis*-acting regulatory elements and to analyse the *BdWRKY* gene promoter sequences.^34^

## 3. Results and discussion

### 3.1. Distribution of WRKY domain-containing proteins in plant kingdom

**Figure 1.**
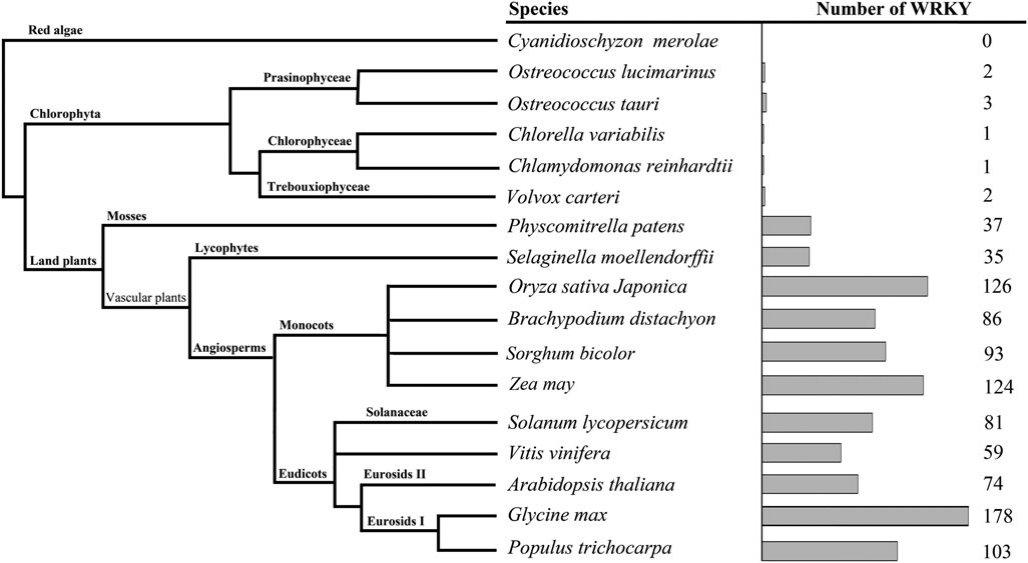
Distribution of the WRKY domain-containing proteins in Plantae. The total number of *WRKY* homologous genes found in each genome is indicated on the right.

WRKY domain-containing proteins are extensively found in plants, some fungi, bacteria, and slime moulds. Here, we searched for *WRKY* genes in six comprehensive datasets, GenBank, UniProt, plantTFDB, GramineaeTFDB, Superfamily, and Phytozome of plant species. In this study, we focused our search and analyses on six major types of model organisms whose genomes have been already sequenced, including red alga, the chlorophytes, the moss, the lycophyte, the eudicots, and the monocots.^35,36^ The result showed that, as a gene super family that plays important roles in regulation of defence response pathways, WRKY TFs conservatively existed in plant kingdom (Fig. 1). In general, only a few of *WRKY* homologous genes could be found in algae genome, while plants possess a large number of *WRKY* genes (Fig. 1). The results indicated that the earliest evolutionary origin of the gene containing the WRKY was from unicellular green algae of chlorophyta, suggesting that WRKY proteins arose before plants transitioned from water to land. With the evolution of species, the land plants have developed a series of highly sophisticated mechanisms that help them to adapt to changing environmental conditions,^37^ and hence, the number of WRKY TFs increased and they were extensively found in land plants in response to the environmental stimuli and regulation of physiological reactions.

### 3.2. Chromosomal distribution and duplication events of *BdWRKY* genes

**Figure 2.**
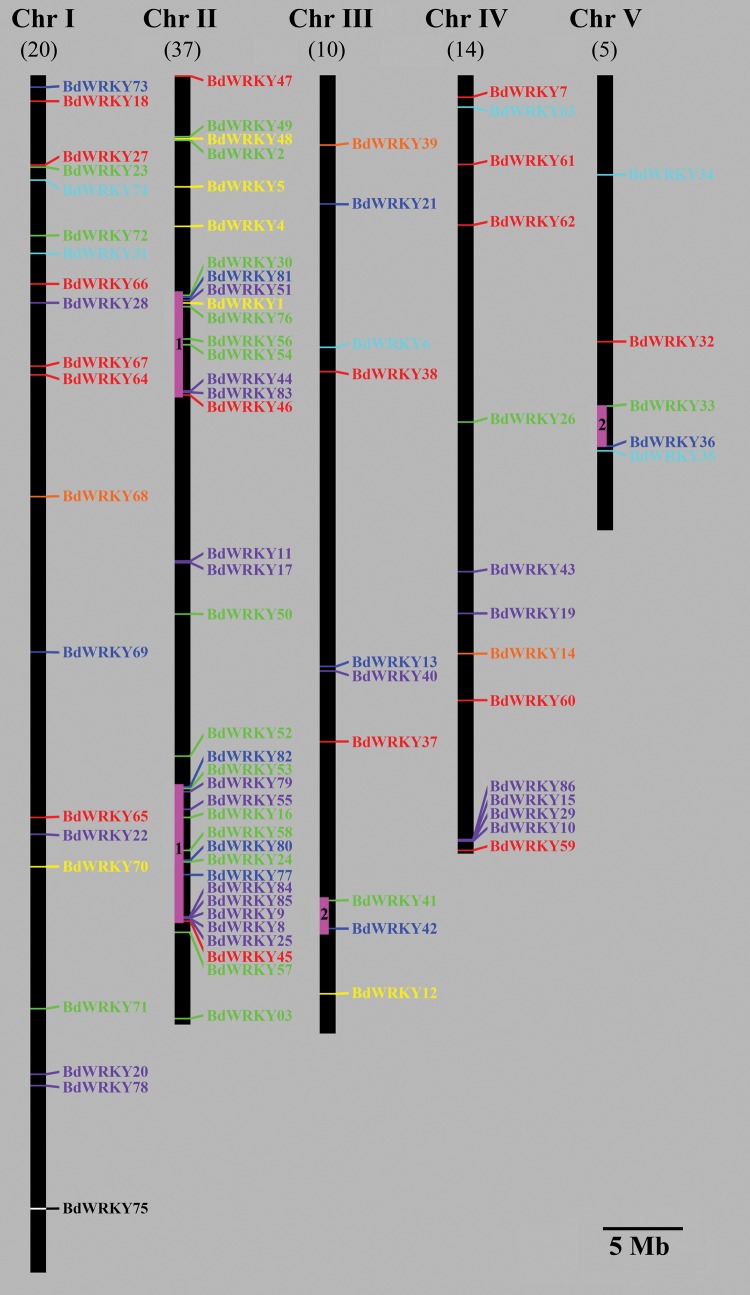
Chromosomal locations and regional duplication for *B. distachyon WRKY* genes. The chromosomal position of each *BdWRKY* was mapped according to the *B. distachyon* genome. The chromosome number is indicated at the top of each chromosome. The number below indicates the number of *BdWRKYs* in each chromosome. The scale is 5 Mb. The bars with numbers on the chromosomes indicate the four predicted duplication regions.

To date, the information regarding expansion events of the *BdWRKY* gene family in *B. distachyon* remains unclear. To investigate the relationship between genetic divergence and gene duplication within the *BdWRKY* gene family in *B. distachyon*, we determined the chromosomal locations of *BdWRKYs* based on the information from the *B. distachyon* genomic database (http://www.brachypodium.org/). The result showed that the *BdWRKYs* were distributed throughout all the five *B. distachyon* chromosomes, most *BdWRKYs* were distributed on Chromosomes 1 and 2 (Fig. 2). Then, the distribution appeared to be uneven. Relatively high densities of *BdWRKYs* were observed on the top and at the bottom arms of Chromosome 2. In contrast, low densities were detected in Chromosomes 3, 4, and 5. Subsequently, we analysed the gene cluster expansion events of *BdWRKYs* in the *B. distachyon* genome. Based on the phylogenetic relationship and sequence similarity, we identified 15 pairs of *BdWRKY* genes with high levels of protein sequence similarity. For instance, the entire protein sequences of *BdWRKY33* and *BdWRKY41* shared 71% similarity, whereas those of *BdWRKY81* and *BdWRKY82* shared 64% similarity. Among *BdWRKY* genes with a high degree of homology, 8 (53%) pairs of *BdWRKYs* reside within chromosomal segments that have clear relatives in the *B. distachyon* genome, suggesting that they may have evolved from duplication events. As shown in Fig. 2, two of those multiple pairs of duplicated regions were located at Chromosome 2, and the others distributed on Chromosomes 3 and 5 (Fig. 2, bars with numbers).

In general, plants can integrate alternative developmental pathways during evolution, and then choose suitable pathways in their growth and development in response to different environmental cues.^38^ It is believed that multiple members of a specific gene family that form a large regulative network to control complicated physiological processes were a result of the long evolutionary history of a particular species.^39,40^ The individual members of a gene family represent a succession of genomic rearrangements and expansions during the process of evolution.^41^ In this study, we found at least four putative segmental duplication events in the *B. distachyon* genome; and those duplications influenced the distribution of *BdWRKY* genes in *B. distachyon*. Particularly, the putative duplications between *BdWRKY33/36* and *BdWRKY41/42* were highly similar. Moreover, on the putative segmental duplications of the Chromosome 2, the order of the *BdWRKY* genes (including *BdWRKY30/81/51/56/46*) arrangement on the top arm of Chromosome 2 was similar to those of the *BdWRKY* genes (including *BdWRKY82/53/16/58/45*) at the bottom arm of this chromosome. *BdWRKY33* and *BdWRKY36* are on a segment of Chromosome 5, and this segment is likely a duplicate of a segment on Chromosome 3 where *BdWRKY41* and *BdWRKY42* are located (Fig. 2). The motif structure of BdWRKY33 is identical with BdWRKY41, while there were only a few differences between BdWRKY36 and BdWRKY42, suggesting that the C-terminal of the segmental duplications on Chromosomes 3 and 5 might diverge to perform new functions during the process of evolution (Fig. 2). Thus, it is inferred that the new gene initially resulted from the duplication, and thereafter diverge from a series of synonymous and/or non-synonymous mutations.

### 3.3. Characteristics of BdWRKY domains

The gene family of TFs usually contain highly conserved domain or domains involved in DNA binding.^42^ Assigning structural domains to protein sequences is important in performing a comprehensive analysis of highly divergent sequences in large gene families.^43^ Based on both the number of WRKY domains and the features of their zinc finger-like motif, the BdWRKY can be classified into three main groups, consistent with the previous report.^29^ The WRKY TFs with two WRKY domains belong to the Group I, while most proteins with one WRKY domain belong to the Group II (Supplementary Fig. S2). Generally, Group I and Group II WRKY TFs share the same type of zinc finger-like motif with a C_2_H_2_ zinc ligand (C–X_4–5_–C–X_22–23_–H–X_1_–H; Supplementary Fig. S2). There is a small subset of BdWRKY TFs containing a C_2_HC motif (C–X_7_–C–X _23_–H–X_1_–C; Supplementary Fig. S2), and this subset is assigned to Group III. Although the WRKYGQK heptapeptide sequence was highly conserved in BdWRKY TFs, sequence similarity beyond the domains is quite low among most genes. As we know, a protein domain is considered as an evolutionary unit of protein function and the domain coding sequence can be duplicated and/or recombined.^44^ From recent research on genomes analysis, new protein functionalities appear to arise from the addition or exchange of protein domains by duplicating one or more domains, recombining fragments of DNA from different organisms, and diverging duplicated sequences by base substitutions, deletions, and insertions.^41,45^ Therefore, the whole family of BdWRKY TFs, which might result from long-time evolutionary history, represented divergent WRKY domains, even in much closely related gene pairs, such as *BdWRKY33/41*, *BdWRKY24/54*, *BdWRKY81/82*, and so on.

**Figure 3.**
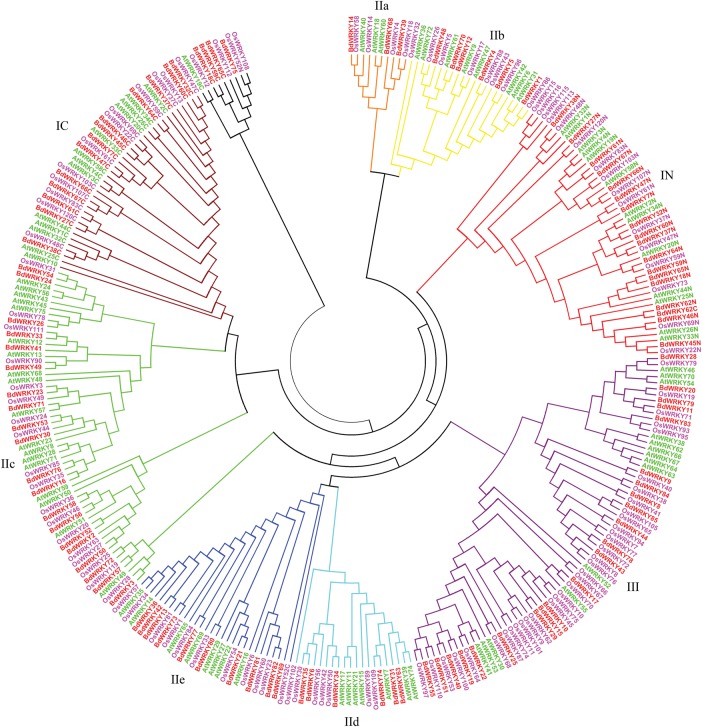
NJ analyses of 301 WRKY domains from *O. sativa*, *A. thaliana*, and *B. distachyon*, containing 262 plant WRKY proteins. The domains clustered into eight major subgroups, IN, IC, IIa, IIb, IIc, IId, IIe, and III.

To further investigate the evolutionary relationships among the WRKY domains from different species, we estimated the phylogeny by using the NJ program from MEGA 5 for the WRKY domains from *O. sativa*, *A. thaliana*, and *B. distachyon*. All subgroups were present in monocots and eudicots (Fig. 3), indicating that the appearance of most WRKY TFs in plants predates the divergence of monocot/eudicots. Meanwhile, no species-speciﬁc subgroups and/or clades were observed in *O. sativa*, *A. thaliana*, or *B. distachyon*, implying that WRKY family genes were more conserved during evolution. In addition, WRKY domains from the same lineage tend to cluster together in the phylogenetic tree, suggesting that they experienced duplications after the lineages diverged (Fig. 3). Furthermore, WRKY phylogenetic tree showed almost the same clustering patterns in *O. sativa* and *B. distachyon* (Fig. 3 and Supplementary Table S3). In total, about 62 pairs of WRKY domains from *O. sativa* and *B. distachyon* were clustered as pairs, indicating that they might be the orthologous WRKY domains (Fig. 3). For example, the WRKY domains of BdWRKY54 and OsWRKY31 are highly similar, indicating that some consensus in domain may have existed before the divergence of *B. distachyon* and *O. sativa*. Meanwhile, only two pairs of WRKY domains from *B. distachyon* and *A. thaliana* could be clustered as pairs, suggesting that the BdWRKY domains are evolutionarily more closely related to OsWRKY domains, which is consistent with the notion that both *B. distachyon* and *O. sativa* belong to monocots. The phylogenetic similarity found in *O. sativa* and *B. distachyon* WRKY domain suggests that they may have evolved conservatively.

### 3.4. Protein structure and tissue-specific expression pattern of *BdWRKY*

**Figure 4.**
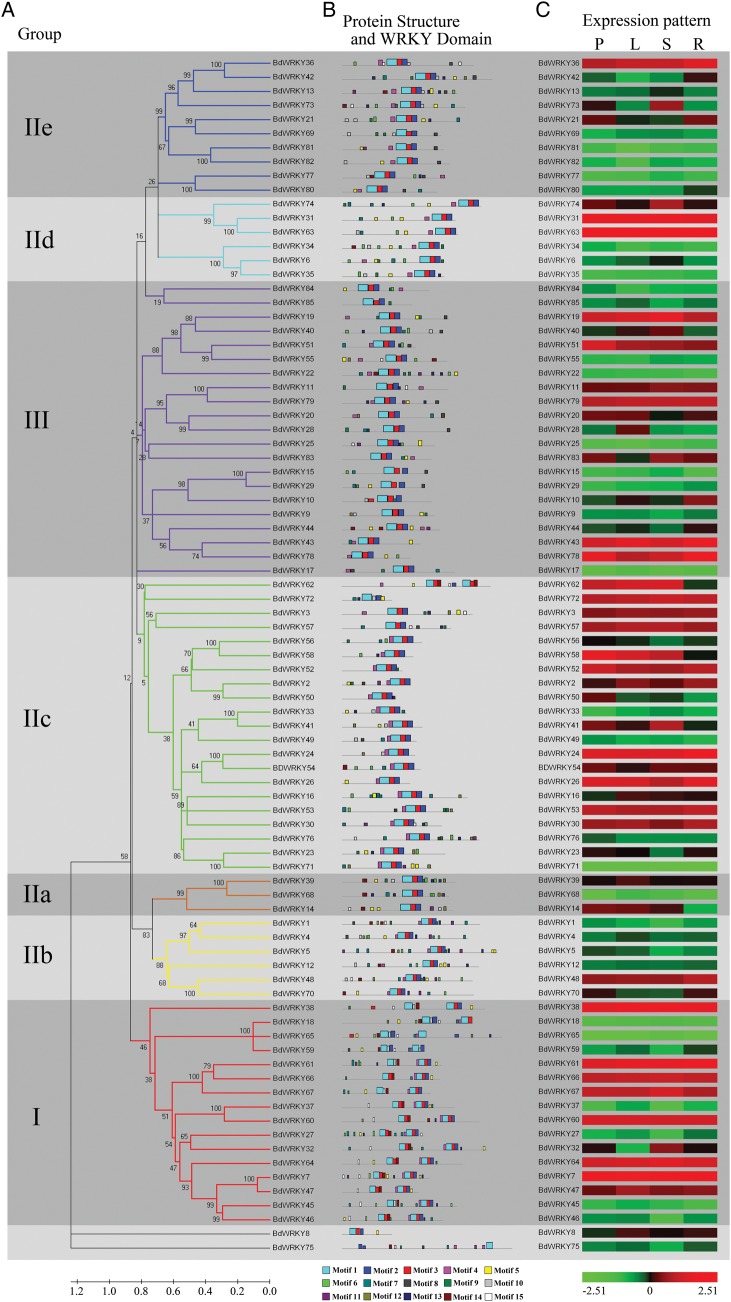
Phylogenetic relationships and subgroup designations in WRKY proteins with tissue-specific expression profile from *B. distachyon*. (A) The phylogenetic tree was constructed from the amino acid sequences using the NJ program from MEGA 5, representing relationships among 86 WRKY proteins from *B. distachyon*. The proteins are clustered into seven subgroups, which are designated with a subgroup number (e.g. IIe) and marked with a different background to facilitate subfamily identification with a high predictive value. The numbers beside the branches represent bootstrap support values (>50%) from 1000 replications. (B) Structure of WRKY proteins and the WRKY domains in *B. distachyon*. The details of sequence logo of motifs were shown in Supplementary Fig. S1. (C) Expression patterns of *WRKY* genes in *B. distachyon* in different tissues. P for seedling, L for leaf, S for stem, R for root. In this expression pattern analysis, the 2-week-old seedlings were used for harvesting different tissues including leaf, stem, and root. The *BdWRKY* array was constituted of 86 primer sets representing all members of the *B. distachyon WRKY* gene family. The expression values of the 86 *BdWRKY* genes were assessed upon the qPCR result analysis.

Based on sequence similarity and protein structure, we also divided the 86 members of the BdWRKY TFs into seven subgroups (I, IIa–e, and III) (Fig. 4A and B). Remarkably, the WRKY domains were almost identical, even though the lengths of the coding region of the *WRKY* genes were different, and the cluster result based on whole BdWRKY sequences was different from the clustering based on BdWRKY domains. A schematic representing the structure of all members of BdWRKY TFs was constructed from the MEME motif analysis results (Fig. 4B).^32^ Most members of the BdWRKYs shared three motifs, Motif 3, Motif 2, and Motif 1 linked in order. A few members, such as BdWRKY8, BdWRKY65, BdWRKY72, and BdWRKY75, showed quite different protein structures compared with other members (Fig. 4B). Interestingly, many of the motifs were selectively distributed among the specific clades in the phylogenetic tree, for example, Motif 8 in Group IIe, and Motif 15 in Group I. The clustered BdWRKY pairs, i.e. BdWRKY81/82, BdWRKY31/63, showed highly similar motif distribution (Fig. 4B). The motifs and their arrangement in the BdWRKYs are similar among proteins within subfamilies, demonstrating that the protein architecture is remarkably conserved within a specific subfamily. The biological functions of many WRKYs remain to be elucidated. The above findings may facilitate the identification of functional units in BdWRKYs and lead to the discovery of their roles in plant growth and development.

TFs usually harbour many types of DNA-binding domains and they can be grouped into a handful of different, often large, gene families.^46^ By forming intricate networks, TFs control the expression of genes in a genome at the transcriptional level.^47^ It has been noted previously that many TF gene families exhibit great disparities in abundance among different organisms and different tissues to exert different physiological functions. Thus, gene expression patterns can provide important clues for gene function. To further analyse the tissue specificity of the *WRKY* gene family members, we confirmed their transcription levels in three different tissues, leaves, stems, and roots. The expression of all of the *WRKY* gene family members was detected in all three tissues (Fig. 4C). The results revealed that several *BdWRKY* genes, including *BdWRKY7*, −*24*, −*31*, −*38*, −*61*, and −*64*, showed higher expression levels than other members of the WRKY family in all the tissues tested. The expression of *BdWRKY78* was highly induced in the root while its expression level was relatively low in the leaf and stem. *BdWRKY32*, −*41*, −*73*, and −*74* showed higher expression levels in the stem than that in the leaf and root (Fig. 4C). The expression pattern of these genes suggested that *BdWRKYs* were involved in the growth and development of organs or tissues under specific conditions. Interestingly, most of clustered gene pairs showed the same expression pattern, such as *BdWRKY31/63*, *BdWRKY81/82*, *BdWRKY77/80*, and so on. On the other hand, gene pairs *BdWRKY37/60*, *BdWRKY36/42*, *BdWRKY56/58*, and other clustered pairs exhibited different expression patterns (Fig. 4C). These results indicated that most of clustered gene pairs had more similarities in the WRKY domain and shared similar expression patterns; they might be functionally redundant. The *BdWRKY* pairs that showed different expression levels may be involved in different signalling pathways. Since the expression of genes was regulated by a series of TFs, the disparities in abundance of *BdWRKY* gene among different tissues suggested that the *BdWRKY* genes, although are TFs themselves, were also regulated by other TFs in different tissues.

### 3.5. Expression profiles of *BdWRKY* upon multiple phytohormone treatments and abiotic or biotic stresses

**Figure 5.**
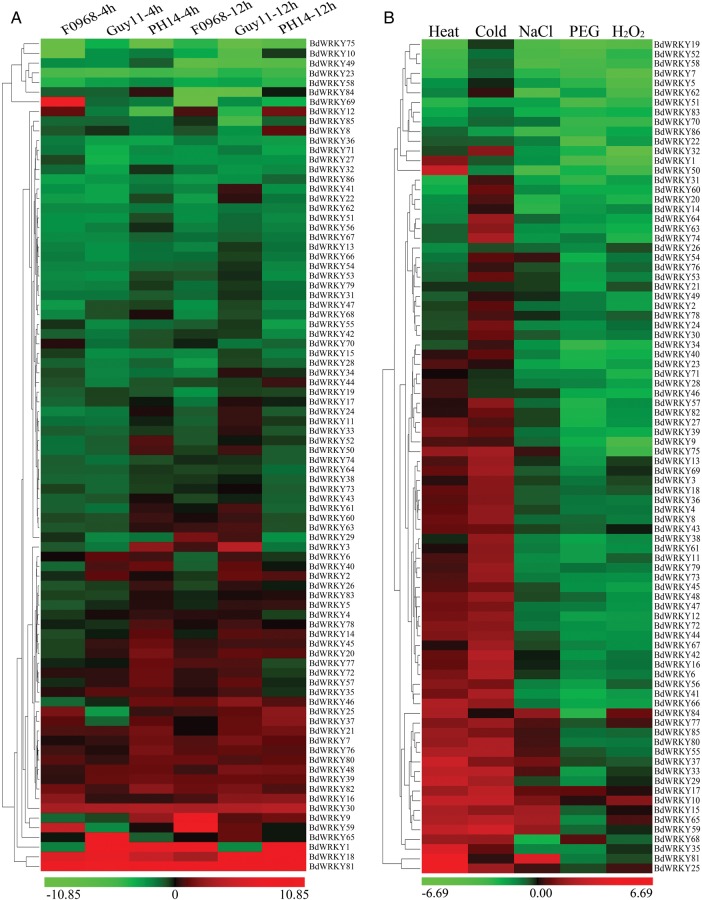
Expression profiles of *BdWRKY* genes under biotic and abiotic stresses. (A) The 2-week-old seedlings were sprayed with different pathogens. (B) Clustering of *BdWRKY* genes according to their expression profiles in the seedling of *B. distachyon* after different stress treatments. The *BdWRKY* array was constituted of 86 primer sets representing all members of the *B. distachyon WRKY* gene family. The expression of the 86 *BdWRKY* genes was assessed upon the qPCR result analysis.

It has been demonstrated that *WRKY* genes were not only involved in the activation of plant defence systems,^48^ but also played key roles in the control of plants' response to environmental stimuli.^3^ Since it has been thought that *BdWRKY* genes are responsive to plant defence-related phytohormones, we investigated the expression profiles of the *WRKY* family genes in *B. distachyon* after phytopathogen treatments. A total of three phytopathogens, including *F. graminearum* (F0968) and two strains of *M. grisea* (Guy11, avirulent ACE1 genotype; PH14, virulent ACE1 genotype), were used to inoculate Bd21 seedling in this study. The expression profiles of the *BdWRKY* family genes at 4 hpi (hour post-inoculation) and 12 hpi were shown in Fig. 5A. The data demonstrated that a large number of *BdWRKY* genes were rapidly and significantly up-regulated after inoculation of phytopathogen within 4 h. At least 15 *BdWRKY* genes were up-regulated by all three phytopathogens treated, while nine *BdWRKY* genes were induced after single phytopathogen inoculation, such as *BdWRKY8*, −*34*, −50, −*69*, −*70*, and so on. As shown in Fig. 5A, the expression levels of *BdWRKY21*, −*37*, −*69*, and −*70* increased remarkably at 4 hpi, and decreased at 12 hpi after F0968 treatment. However, several *BdWRKY* genes (*BdWRKY1*, −*9*, −*29*, etc.) were up-regulated 12 h after F0968 inoculation. These data suggested that *BdWRKY21*, −*37*, −*69*, and −*70* were the early response TFs upon phytopathogen F0968 attack, while *BdWRKY1*, −*9* and −*29* were induced at the second stage of the F0968 infection. Interestingly, numbers of *BdWRKY* genes (e.g. *BdWRKY3*, −*72* and -*77*) were induced faster by PH14 than by Guy11. They were up-regulated at 4 hpi after infection by PH14, but at 12 hpi, they were down-regulated by PH14 and up-regulated by Guy11. Since the pathogenic ability of virulent ACE1 genotype (PH14) was stronger than the wild-type Guy11, these results suggested that the expression of *BdWRKY* genes were very sensitive to biotic stress and the regulation of BdWRKYs were important to plant defence. *BdWRKY* as TF genes were first induced or repressed by phytopathogen, and then, were involved in the regulation of plant defence gene expression.

Similarly, the expression profiles of the *BdWRKY* family genes under different stress conditions were also examined using the qRT-PCR in our study. A total of five stress types, i.e. heat, cold, NaCl, PEG, and H_2_O_2_, were tested in this study. Detailed expression profiles of the *WRKY* family genes under different stress conditions were provided in Supplementary Table S4. Heatmap representation of expression profiles of these *WRKY* family genes under different stress conditions are shown in Fig. 5B. The data revealed that 60 and 80% of *BdWRKY* genes were up-regulated under heat and cold stress conditions, respectively. More than 50% of the *BdWRKY* genes were up-regulated under more than one stress conditions. For examples, *BdWRKY10*, −*33*, −*59*, and −*65* were up-regulated in both heat and cold treatments, while *BdWRKY81* showed a high up-regulation under heat and salt stresses. It has been reported that the severity of the stress and the metabolic status of the plant affected the capacity of plant to tolerate abiotic stress.^49^ ABA as a phytohormone plays an important role in integrating various abiotic or biotic stress signals and controlling downstream stress responses.^49^ Here, our data indicated that almost 50% of the *BdWRKY* genes were down-regulated under three or more stress conditions, which is consistent with the results of most *BdWRKY* gene down-regulated by ABA treatment. For example, *BdWRKY19*, −*22*, −*51*, and −*52* were down-regulated by PEG (drought stress), and similarly, their expression levels were very low after ABA treatment. These correlations of *BdWRKY* genes expression levels between abiotic stress and phytohormone treatment suggest that BdWRKY regulation of downstream gene expression may be linked to stress-induced phytohormone alteration.

**Figure 6.**
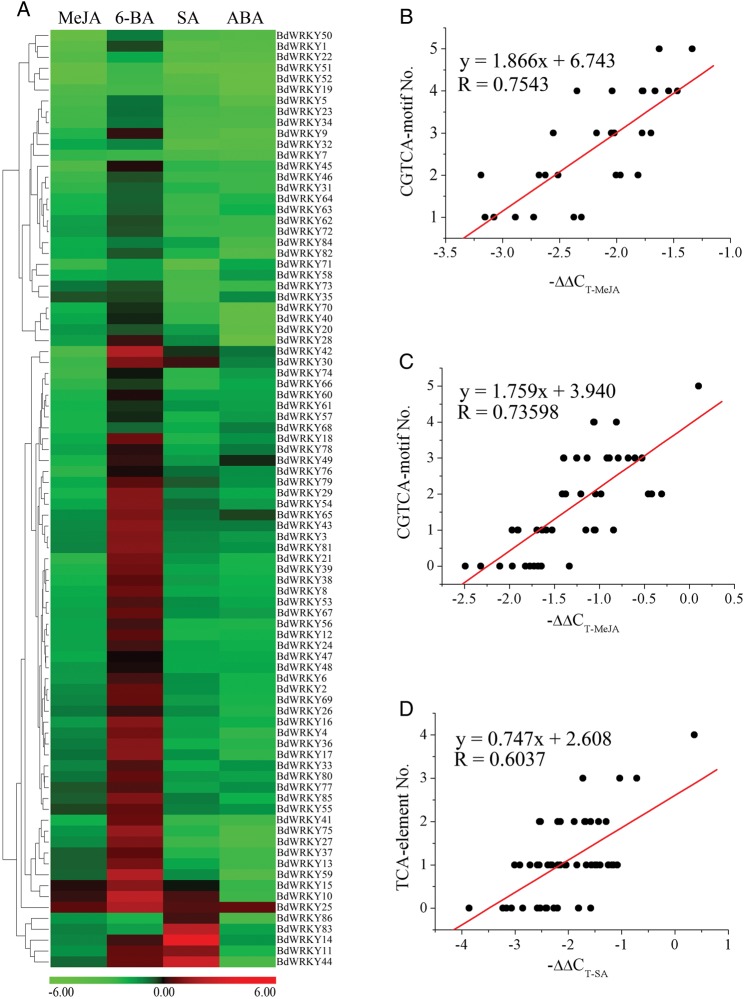
The expression profiles of *BdWRKY* genes under hormone treatment. (A) Clustering of *BdWRKY* genes according to their expression profiles in the seedling of *B. distachyon* after different phytohormone treatments. (B and C) The relevance analysis between MeJA-related elements and MeJA-induced *BdWRKY* gene expression. (D) The relevance analysis between SA-related elements and SA-induced *BdWRKY* gene expression.

Recent studies of the *OsWRKY* genes have also shown that many of *OsWRKY* genes were responsive to JA, SA, and ABA treatments.^50,51^ It has also been reported that WRKY TFs were key factors for the increased transcript abundance of SA- and JA-responsive genes.^52–54^ To investigate the hormonal control mechanisms underlying *BdWRKY* gene expression, we treated Bd21 seedlings with four phytohormones, MeJA, SA, 6-BA, and ABA, respectively and analysed the changes in transcript abundance of these 86 *BdWRKY* genes using qRT-PCR. Our results demonstrated that most of *BdWRKY* genes were repressed by ABA after 3 h of treatment (Fig. 6A). In contrast, 52 of the 86 *BdWRKY* genes were up-regulated within 3 h treatment of 6-BA. Only three and eight *BdWRKY* genes exhibited increased expression levels in response to MeJA and SA treatments, respectively. Seven of 86 *BdWRKY* genes exhibited positive modulation of expression in response to two phytohormones, while only *BdWRKY25* showed up-regulation in response to all four phytohormones. Interestingly, *BdWRKY14* showed very high expression after SA treatment, even though its expression level was decreased after MeJA or ABA treatment. It has been reported that the expression of two genes, *OsWRKY45* and −*62* (*OsWRKY71* and −*14* in this paper, respectively), were increased in SA (SA)-treated rice leaves.^50^ Similarly, *BdWRKY11* and −*14* (the homologous genes of *OsWRKY45* and −*62*) also showed a rapid increase after SA treatment. The results indicated that some homologous genes between *O. sativa* and *B. distachyon* share functional conservation. On the other hand, our data also revealed that a number of *B. distachyon* homologous of rice WRKY genes (e.g. *OsWRKY48*/*BdWRKY38*) did not show similar expression patterns, suggesting that the functions of some genes were altered during the evolution. According to the statistical analysis, there is a good correlation between the number of MeJA-inducible *cis*-element and the expression levels in most *BdWRKY* genes after 3 h MeJA treatment (Fig. 6B and C, and Supplementary Fig. S4). Similarly, the number of SA-inducible *cis*-element showed a good correlation with the expression levels of most *BdWRKY* genes after 3 h SA treatment (Fig. 6D). These results indicated that BdWRKY TFs were regulated by exogenous phytohormones and then bind to the W-box in promoters of downstream genes and regulate their expressions.

## Supplementary data

Supplementary data are available at www.dnaresearch.oxfordjournals.org.

## Funding

This work was supported by grant for Starting Package to the Research Group of Plant Abiotic Stress and Plant Genome Evolution in Shanghai Chenshan Plant Science Research Centre, Chinese Academy of Sciences, and Shanghai Chenshan Botanic Garden from Shanghai Landscaping Administrative Bureau (No. F0112423 and F0122415), the Fund for National Key Laboratory of Plant Molecular Genetics (Y109Z11161), and the Special Fund for Chinese Academy of Sciences (CZBZX-1).

## Supplementary Material

Supplementary Data
