# Supplementary material for: Genome-Wide Evolutionary Characterization and Expression Analyses of WRKY Family Genes in Brachypodium distachyon
Source: DNA Res. 2014 Jan 21;21(3):327–39. doi: 10.1093/dnares/dst060 (PMC4060952; doi:10.1093/dnares/dst060)
Supplement: Supplementary Data [file supp_21_3_327__index.html]

Genome-Wide Evolutionary Characterization and Expression Analyses of WRKY Family Genes in Brachypodium distachyon — Genome-Wide Evolutionary Characterization and Expression Analyses of WRKY Family Genes in Brachypodium distachyon — Supplementary Data 

# Genome-Wide Evolutionary Characterization and Expression Analyses of WRKY Family Genes in *Brachypodium distachyon*

## Supplementary Data

Supplementary Data

**Files in this Data Supplement:**

- Supplementary Tables - pdf file
- Supplementary Figure 1 - tif file
- Supplementary Figure 2 - tif file
- Supplementary Figure 3 - tif file
- Supplementary Figure 4 - tif file
