# Supplementary material for: Genome-Wide Evolutionary Characterization and Expression Analyses of WRKY Family Genes in Brachypodium distachyon
Source: DNA Res. 2014 Jan 21;21(3):327–39. doi: 10.1093/dnares/dst060 (PMC4060952; doi:10.1093/dnares/dst060)
Supplement: Supplementary Data [file supp_dst060_dst060supp_tables.pdf]

Supplementary Table 1. The WRKY transcription factor family in *B. distachyon*.

Gene Name	Location	Gene Model	Comments
**BdWRKY1**	Bd2:14220256..14222873	Bradi2g16150.1	Group IIb
**BdWRKY2**	Bd2:3983627..3984262	Bradi2g05510.1	Group IIc
**BdWRKY3**	Bd2:58924622..58926999	Bradi2g62130.1	Group IIc
**BdWRKY4**	Bd2:9403194..9408009	Bradi2g11170.1	Group IIb
**BdWRKY5**	Bd2:6965062..6969356	Bradi2g08620.1	Group IIb
**BdWRKY6**	Bd3:17079694..17080883	Bradi3g18580.1	Group IId
**BdWRKY7**	Bd4:1276705..1278741	Bradi4g01950.1	Group I
**BdWRKY8**	Bd2:52655598..52657803	Bradi2g53510.1	-
**BdWRKY9**	Bd2:52646076..52649410	Bradi2g53500.1	Group III
**BdWRKY10**	Bd4:47804750..47805919	Bradi4g44370.1	Group III
**BdWRKY11**	Bd2:30388047..30389483	Bradi2g30695.1	Group III
**BdWRKY12**	Bd3:57392174..57394564	Bradi3g57710.1	Group IIb
**BdWRKY13**	Bd3:36924565..36925829	Bradi3g34567.1	Group IIe
**BdWRKY14**	Bd4:36119270..36124180	Bradi4g30360.1	Group IIa
**BdWRKY15**	Bd4:47786980..47788750	Bradi4g44350.1	Group III
**BdWRKY16**	Bd2:46341174..46344106	Bradi2g45900.1	Group IIc
**BdWRKY17**	Bd2:30398035..30400975	Bradi2g30800.1	Group III
**BdWRKY18**	Bd1:1580840..1582503	Bradi1g02327.1	Group I
**BdWRKY19**	Bd4:33637416..33639506	Bradi4g28280.1	Group III
**BdWRKY20**	Bd1:62421625..62422845	Bradi1g63220.1	Group III
**BdWRKY21**	Bd3:8007304..8009052	Bradi3g09810.1	Group IIe
**BdWRKY22**	Bd1:47430434..47433797	Bradi1g48770.1	Group III
**BdWRKY23**	Bd1:5717785..5722044	Bradi1g08106.1	Group IIc
**BdWRKY24**	Bd2:49173622..49177995	Bradi2g49020.1	Group IIc
**BdWRKY25**	Bd2: 52664751 - 52666466	Bradi2g53520.1	Group III
**BdWRKY26**	Bd4:21655844..21659743	Bradi4g19060.1	Group IIc
**BdWRKY27**	Bd1:5619653..5623406	Bradi1g07970.1	Group I
**BdWRKY28**	Bd1:14207355..14209349	Bradi1g17660.1	Group III
**BdWRKY29**	Bd4:47797837..47799556	Bradi4g44360.1	Group III
**BdWRKY30**	Bd2:13707176..13708809	Bradi2g15360.1	Group IIc
**BdWRKY31**	Bd1:11188915..11191904	Bradi1g14300.1	Group IId
**BdWRKY32**	Bd5:16639846..16643118	Bradi5g13090.1	Group I
**BdWRKY33**	Bd5:20674827..20679112	Bradi5g17395.1	Group IIc
**BdWRKY34**	Bd5:6203333..6204805	Bradi5g04817.1	Group IId
**BdWRKY35**	Bd5:23482100..23483388	Bradi5g20700.1	Group IId
**BdWRKY36**	Bd5:23193818..23196993	Bradi5g20290.1	Group IIe
**BdWRKY37**	Bd3:41662537..41665191	Bradi3g39340.1	Group I
**BdWRKY38**	Bd3:18514515..18520937	Bradi3g19640.1	Group I
**BdWRKY39**	Bd3:4354442..4356174	Bradi3g06070.1	Group IIa
**BdWRKY40**	Bd3:37214494..37216071	Bradi3g34850.1	Group III
**BdWRKY41**	Bd3:51584113..51587575	Bradi3g50360.1	Group IIc

**BdWRKY42**	Bd3:53316886..53321459	Bradi3g52420.1	Group IIe
**BdWRKY43**	Bd4:31073069..31076118	Bradi4g25717.1	Group III
**BdWRKY44**	Bd2:19779563..19782548	Bradi2g22230.1	Group III
**BdWRKY45**	Bd2:52860060..52862718	Bradi2g53760.1	Group I
**BdWRKY46**	Bd2:19960366..19962573	Bradi2g22440.1	Group I
**BdWRKY47**	Bd2:37371..39452	Bradi2g00280.1	Group I
**BdWRKY48**	Bd2:3979821..3981719	Bradi2g05500.1	Group IIb
**BdWRKY49**	Bd2:3818524..3823413	Bradi2g05234.1	Group IIc
**BdWRKY50**	Bd2:33674703..33675549	Bradi2g33540.1	Group IIc
**BdWRKY51**	Bd2:14040691..14041846	Bradi2g15877.1	Group III
**BdWRKY52**	Bd2:42525077..42527076	* LOC100843345	Group IIc
**BdWRKY53**	Bd2:44578672..44580858	Bradi2g44090.1	Group IIc
**BdWRKY54**	Bd2:16801079..16803652	Bradi2g19070.1	Group IIc
**BdWRKY55**	Bd2:45857664..45858974	Bradi2g45480.1	Group III
**BdWRKY56**	Bd2:16474142..16475652	Bradi2g18530.1	Group IIc
**BdWRKY57**	Bd2:53553539..53556063	Bradi2g54720.1	Group IIc
**BdWRKY58**	Bd2:48426948..48428121	Bradi2g48090.1	Group IIc
**BdWRKY59**	Bd4:48418469..4842114	Bradi4g45290.1	Group I
**BdWRKY60**	Bd4:39074575..39078767	Bradi4g33370.1	Group IIc
**BdWRKY61**	Bd4:5576052..5581378	Bradi4g06690.1	Group I
**BdWRKY62**	Bd4:9350003..9357060	Bradi4g09890.1	Group I
**BdWRKY63**	Bd4:1992609..1995531	Bradi4g02680.1	Group IId
**BdWRKY64**	Bd1:18715369..18721632	Bradi1g23340.1	Group I
**BdWRKY65**	Bd1:46394072..46396455	Bradi1g47690.1	Group I
**BdWRKY66**	Bd1:13042696..13049431	Bradi1g16120.1	Group I
**BdWRKY67**	Bd1:18197079..18200281	Bradi1g22680.1	Group I
**BdWRKY68**	Bd1:26329079..26330580	Bradi1g30870.1	Group IIa
**BdWRKY69**	Bd1:36017779..36022778	* LOC100834454	Group IIe
**BdWRKY70**	Bd1:49453669..49457672	Bradi1g51030.1	Group IIb
**BdWRKY71**	Bd1:58314278..58317315	Bradi1g59180.1	Group IIc
**BdWRKY72**	Bd1:10018214..10019283	Bradi1g13207.1	Group IIc
**BdWRKY73**	Bd1:723239..728467	* LOC100845846	Group IIe
**BdWRKY74**	Bd1:6545010..6548310	Bradi1g09170.1	Group IId
**BdWRKY75**	Bd1:70816062..70817256	* LOC100837754	-
**BdWRKY76**	Bd2:14427911..14430851	Bradi2g16357.1	Group IIc
**BdWRKY77**	Bd2:49904315..49905775	Bradi2g49906.1	Group IIe
**BdWRKY78**	Bd1:63118295..63119746	Bradi1g63910.1	Group III
**BdWRKY79**	Bd2:44782012..44783510	Bradi2g44270.1	Group III
**BdWRKY80**	Bd2:49070580..49071586	Bradi2g48907.1	Group IIe
**BdWRKY81**	Bd2:13729497..13730737	Bradi2g15405.1	Group IIe
**BdWRKY82**	Bd2:44527990..44529664	Bradi2g44035.1	Group IIe
**BdWRKY83**	Bd2:19783798..19788797	Bradi2g22240.1	Group III
**BdWRKY84**	Bd2:52617265..52627264	Bradi2g53480.1	Group III
**BdWRKY85**	Bd2: 52632487..52636282	Bradi2g53495.1	Group III

**BdWRKY86**	Bd4:47782844..47784664	Bradi4g44342.1	Group III

* GeneBank Locus ID

Supplementary table 2. The list of primer-sets of BdWRKY genes for qRT-PCR.

Gene Name	Gene Model	Forward Primer	Reverse Primer	Tm-FP	Tm-RP
**BdWRKY1**	Bradi2g16150.1	GCGAGATGGACTTCTTCGAG	TGGCCTCCATGTAGTGATGA	60.1	60.1
**BdWRKY2**	Bradi2g05510.1	ATGGAAGCGGGAAACTTCTG	ATGGAGGAGTAGCAGGCAAA	61.5	59.8
**BdWRKY3**	Bradi2g62130.1	GAGGAATATTGGCAGGACGA	CGAGATGAGCTTGTTCACCA	60	60
**BdWRKY4**	Bradi2g11170.1	GCAATAGGAGGACGGTTCAA	TACTTCCTCCATTGGCATCC	60.1	59.9
**BdWRKY5**	Bradi2g08620.1	CAGGATTCTCGATCCATTCC	CAAGTTGAGGAACCCGAGAG	59.4	59.8
**BdWRKY6**	Bradi3g18580.1	CCGTGACTTCCACATCCTTC	GGAGTTGCTGTTGGAGCTTG	60.5	61
**BdWRKY7**	Bradi4g01950.1	CCTCGCACTTGTCCTCCTAC	AAGAATTTGTTTGGGCGTTG	59.9	60
**BdWRKY8**	Bradi2g53510.1	ACGGGTACATGTGGAGGAAG	TGCTCGTGGACGTAGATGAC	59.8	59.9
**BdWRKY9**	Bradi2g53500.1	CGAAGGCGGGTATAATCTCA	GCGTTTATCTTTGGCTGGAG	60	59.9
**BdWRKY10**	Bradi4g44370.1	ATGGCGGATGACAAGAAGAG	GTAGCTCCTTGGGTGCTTTG	60.2	59.9
**BdWRKY11**	Bradi2g30695.1	AGAAGGACCCAGCAATCCTC	ACCCTGAAGGTGTCCTTGTC	60.6	59
**BdWRKY12**	Bradi3g57710.1	ACAAGGTGAGGTACCGATGG	CGGAGCATCTTGTTCTCCTC	59.8	60
**BdWRKY13**	Bradi3g34567.1	ATCTCTCGGATGTGGTGAGG	GGCATCATCCGTCATAATCC	60.1	60.1
**BdWRKY14**	Bradi4g30360.1	GGGTATCAATGGCGGAAGTA	ACGCAGCTCGAAGTAAGCTC	59.8	59.8
**BdWRKY15**	Bradi4g44350.1	ACCAATGTTCCCCACTACGA	TGCCATTGCTAATGTTCTCG	60.2	59.8
**BdWRKY16**	Bradi2g45900.1	CGATGAAGGTGACAAGAGCA	TTCTTCACCACGCACTTCTG	60	60
**BdWRKY17**	Bradi2g30800.1	CTACCGGTGCACTCACAAGA	GGTTGGAGAATTGGTCGTTG	59.9	60.4
**BdWRKY18**	Bradi1g02327.1	GGTTGCTGAGCTTTTGAAGG	GAGAAGTAATCGGCGTGGAA	60	60.2
**BdWRKY19**	Bradi4g28280.1	CGTCTCCGACATGACTTTCA	TGCTTGGCTCCAAGAATCTC	59.8	60.5
**BdWRKY20**	Bradi1g63220.1	CACGCACAAATCAGACCAAG	TAAAGTCTGCGCGAAACTGA	60.3	59.8
**BdWRKY21**	Bradi3g09810.1	GTGCCGAGATCCAAGAGAAG	GCTGCTGCACCGGTAGTATC	60	60.8
**BdWRKY22**	Bradi1g48770.1	CAAAGCGTGTGCAAGAGAAG	ACCGGTAGTAACCCCTTGGA	59.8	60.6
**BdWRKY23**	Bradi1g08106.1	GAGACTGGCGTTAGCTCGAC	ACTCCAGGAGGCACGATGTC	60.2	62.6
**BdWRKY24**	Bradi2g49020.1	TACCGGTGGAGAAAGTACGG	CGGGAGAGAAACTGAAGCTG	60	60
**BdWRKY25**	Bradi2g53520.1	TTCCTCAACTTGCTATCGCC	AGACCCGGCTCTTCTTCATG	60.4	61.7
**BdWRKY26**	Bradi4g19060.1	GAACAACAACTTCCCCAGGA	TGTGGTTGCTAAAGGCTGTG	59.9	59.9
**BdWRKY27**	Bradi1g07970.1	CCGTCCCAGGAGCTACTACA	CCTTGAGCTGACGAGCAGTA	60.3	59.3
**BdWRKY28**	Bradi1g17660.1	TCCTCATTCCATGCTCTTCC	GCGACGAAGGGAAGCTACTA	60.2	59.6
**BdWRKY29**	Bradi4g44360.1	AGTCCTTGGTGTTGGTGAGG	GCAGCTCCGTTCTTTGTAGG	60	60
**BdWRKY30**	Bradi2g15360.1	AACGGGTTGAAGAAGAAGCA	GCACCGGTAGTAGCTCCTTG	59.9	59.9
**BdWRKY31**	Bradi1g14300.1	GGCTAAACTCCTCAGCAACG	TCGAAGGATGGGCACTATTC	60	60
**BdWRKY32**	Bradi5g13090.1	CAACCACAGGGAAGTTGTTC	ATGCTGGTTGCTGGGTAAAG	58	60.1
**BdWRKY33**	Bradi5g17395.1	ATGGCGTGATGTTTTCTTCC	GCCATGTCTAGTTGCAGTGG	59.9	59.3
**BdWRKY34**	Bradi5g04817.1	GATCTGATGGGAGGATACGG	CTTGAACTTGGACACCGTCA	59.3	59.7
**BdWRKY35**	Bradi5g20700.1	TCCACCTCCTTCTTCTCGTC	CTTCACGCGGTTCTTTCTTC	59.4	60
**BdWRKY36**	Bradi5g20290.1	TGCTCGTCATCACCTACACC	TTGTGCTAGTGGTCGTCGTC	59.7	59.9
**BdWRKY37**	Bradi3g39340.1	CCACCAATTTTTCCAACCAC	TCTCGGTCTGCATCACACTC	60.1	60
**BdWRKY38**	Bradi3g19640.1	AGTGATTGTGAAGGGGATGC	TACGCCATCTGTACCCATCA	59.9	59.9
**BdWRKY39**	Bradi3g06070.1	CGTCGAGGAGAACTTCTTGC	TGGTCATGTCGCTGTACTGG	60.1	60.7
**BdWRKY40**	Bradi3g34850.1	GGGCATAGCTGGAGGAAGTA	CGTGGTAGACGACGTCAAAG	59.3	59.4
**BdWRKY41**	Bradi3g50360.1	AGAAGCCGTAATGCTCTCCA	TTATTAGCACCCTCGCATCC	60	60.1

**BdWRKY42**	Bradi3g52420.1	TCAGAGGACTGCAGATGTCG	CCAGAGATCCGAAGGAACAA	60.1	60.2
**BdWRKY43**	Bradi4g25717.1	AATCTGCTTCGGCTCAAGTG	GATCGACGAAGACGAAGAGC	60.5	60.1
**BdWRKY44**	Bradi2g22230.1	CTTCGATTTCTTCCCCAACA	ACCACTTGAGGCATCCTGAC	60	60.1
**BdWRKY45**	Bradi2g53760.1	TCCGACTTCTCGTTCCAGAC	GAAGCTCGCTGGGTTGTTAG	60.4	60
**BdWRKY46**	Bradi2g22440.1	CAACATGCTCGGAAACAATG	GCTCCACCTTCTTCTTCGTG	60.1	60
**BdWRKY47**	Bradi2g00280.1	GAAAGTACGGGCAGAAGCAG	ACGGGTGGAGAGGTGTTATG	60	59.8
**BdWRKY48**	Bradi2g05500.1	CAACAAGCAGGAGGAGGAAG	TGAGAGGATTTGGGCTTGAC	60	60
**BdWRKY49**	Bradi2g05234.1	TCAAAGGTTGCACTCGATCA	CAGCTGCTTTCTTCATCCTC	60.3	57.8
**BdWRKY50**	Bradi2g33540.1	CGACTCCACCGGTCTTCTTA	CCGAGCAGCGGTAGTAGTTC	60.2	60
**BdWRKY51**	Bradi2g15877.1	ACCTGCAGAGACACGATCAG	GGATCCAGGAACAACAGCTC	59	59.7
**BdWRKY52**	[*] LOC100843345	CAGCAAAGCAGCAAGTTCAG	TCTTGCCGTACTTCCTCCAC	59.9	60.3
**BdWRKY53**	Bradi2g44090.1	TGGCCAAGGATAATGAGGAG	GCACCGGTAGTAGCTCCTTG	60	59.9
**BdWRKY54**	Bradi2g19070.1	AGGGGATCAAAGGGAACAAC	GCACCGGTAGTAGCTCCTTG	60.2	59.9
**BdWRKY55**	Bradi2g45480.1	CTCTTCGACGTCGTGTACCA	CTTATTGGAGGGGAACGACA	59.9	59.9
**BdWRKY56**	Bradi2g18530.1	GTCGCTGCTCTTGGTAGGAG	GGACTTCTTGCCGTACTTGC	60.2	59.9
**BdWRKY57**	Bradi2g54720.1	ACGTAGGAGGAGGAGGAAGC	GCAAGTCGAGGCAATACCAG	59.8	60.8
**BdWRKY58**	Bradi2g48090.1	GACGGGTACAAGTGGAGGAA	CCGGAAGTAGCATCTTGAGC	60	60
**BdWRKY59**	Bradi4g45290.1	CATGGTTTGGGGAGGCTATG	GAGCTCGGCCAAGAAAAGTA	62.5	59.6
**BdWRKY60**	Bradi4g33370.1	TCGACTCTGCATCTTTGTGG	CATGTGTTGCCCTGTCAATC	60	60
**BdWRKY61**	Bradi4g06690.1	TGAGCTCTGCAGCCAATATG	TTGGGCAATCACTACCCTTC	60.1	59.9
**BdWRKY62**	Bradi4g09890.1	CAGGGCACTCAAACAATGTG	ACTCCAAAGGGAATGCAATG	60.1	59.9
**BdWRKY63**	Bradi4g02680.1	AAGATGGTCCAGCCATTGTC	TGTCCAAGCTAGCCACACTG	59.9	60
**BdWRKY64**	Bradi1g23340.1	TTCAAACCAGGGTGAAGACC	GCAGCATCAATACCAGCAGA	59.9	60
**BdWRKY65**	Bradi1g47690.1	TGTGCACCCAATTTTGAGAA	GCGTGGAACCAGTGGTAGTG	60.1	61.6
**BdWRKY66**	Bradi1g16120.1	CATCGACAAGCCTTAGCACA	GTAAGTGATGGTGGGGTTGG	60	60.1
**BdWRKY67**	Bradi1g22680.1	GGAAGGGGGAGATGAGAAAG	TAGCCATCGTCCAAAAGGTC	60	60.1
**BdWRKY68**	Bradi1g30870.1	GTCGAGGAGGACTTCATGTC	TTGTTATTGGCTTCCGCTTG	57.2	61
**BdWRKY69**	[*] LOC100834454	CCCTCATCCTCACCTACACC	TGGTCCTCCATCTCCATCTC	59.4	60
**BdWRKY70**	Bradi1g51030.1	CTCACTCTCACTCGCCCTTC	ACGAGGATCCTGACAACGAC	60.1	60.1
**BdWRKY71**	Bradi1g59180.1	GGCCATCACCTCCTCTACAA	TGTCAACGGCAGGAACTGTA	60.1	60.3
**BdWRKY72**	Bradi1g13207.1	AGTGGAAGAAGTACGGCCAG	TGCAGGAGGAAGACGATGAC	60.1	61.4
**BdWRKY73**	[*] LOC100845846	CTCACCAGCAGTCAGCTCAG	TGGGTTTCTGGCCATACTTC	59.9	59.9
**BdWRKY74**	Bradi1g09170.1	AGCCTCCAAGATCACCAATG	GACTAGTGTTCGGGCTCAGG	60.1	59.9
**BdWRKY75**	[*] LOC100837754	GTGTGTGGACTATCGCCACC	ATGCCCACTGTGTGATTTGA	61.1	60
**BdWRKY76**	Bradi2g16357.1	CAACAATGGGAAAGGGAAGA	GCACCGGTAGTAGCTCCTTG	59.9	59.9
**BdWRKY77**	Bradi2g49906.1	GCTCGTCACCTACTCCTTCG	GTTCCTGATCCTGCTCTTGC	60	60
**BdWRKY78**	Bradi1g63910.1	TTACCCCCACAGAGTTCCAG	TCAGTGCAGACCTGCTTTTG	60	60.2
**BdWRKY79**	Bradi2g44270.1	GATCGGAGGTGCAGAGAGAG	CCGAGTTCTGGATGTCCTTC	60.1	59.7
**BdWRKY80**	Bradi2g48907.1	CCTACGAGCACAACCACTCA	CGTAGAGCAGCAGGTCGTC	59.9	59.7
**BdWRKY81**	Bradi2g15405.1	GCAGGAAGAGCCAAGTGAAG	GCAGTGGTCAGCAATGTAGG	60.1	59.3
**BdWRKY82**	Bradi2g44035.1	ATTTGGATCACAAGCCGTTC	CCACCTTCTTCAGCTGGCTC	59.9	62.4
**BdWRKY83**	Bradi2g22240.1	GGACGGGTACATATGGAGGA	GCGGCTGTGGTGGTAGTAAC	59.6	60.6
**BdWRKY84**	Bradi2g53480.1	TTCGATGGAGCCAACAAAAG	TTGCCCCTGATCATCTTCTG	61.1	61.1

**BdWRKY85**	Bradi2g53495.1	CCATCATCACGAGCATCATC	ATTCAGCCCGTATCTCAACG	60	60.1
**BdWRKY86**	Bradi4g44342.1	TTGCTGGGATGCAAGTACAG	TTGGCAGCTGTTGAGAACAC	59.8	60
**BdActin**	Bradi2g24070.1	CCCGATGGACAGGTTATCACTA	ATAGAGCCACCAATCCAAACAC	61.1	60.2

* GeneBank Locus ID

Supplementary table 3. The WRKY domain sequences from *O. sativa, A. thaliana* and *B. distachyon*.

name	gene model	WRKY domain
AtWRKY1N	At2g04880	IREKVMEDGYNWRKYGQKLVKGNEFVRSYYRCTHPNCK AKKQLERSAGGQVVDTVYFGEHDHPKP
AtWRKY1C	At2g04880	TLFDIVNDGYRWRKYGQKSVKGSPYPRSYYRCSSPGCPV KKHVERSSHDTKLLITTYEGKHDHDMP
AtWRKY2N	At5g56270	AGGAPAEDGYNWRKYGQKLVKGSEYPRSYYKCTNPNCQ VKKKVERSREGHITEIIYKGAHNHLKP
AtWRKY2C	At5g56270	SDVDILDDGYRWRKYGQKVVKGNPNPRSYYKCTAPGCT VRKHVERASHDLKSVITTYEGKHNHDVP
AtWRKY3N	At2g03340	NADKPADDGYNWRKYGQKQVKGSDFPRSYYKCTHPACP VKKKVERSLDGQVTEIIYKGQHNHELP
AtWRKY3C	At2g03340	SEVDLLDDGYRWRKYGQKVVKGNPYPRSYYKCTTPDCG VRKHVERAATDPKAVVTTYEGKHNHDVP
AtWRKY4N	At1g13960	NVDKPADDGYNWRKYGQKQVKGSEFPRSYYKCTNPGCP VKKKVERSLDGQVTEIIYKGQHNHEPP
AtWRKY4C	At1g13960	SEVDLLDDGYRWRKYGQKVVKGNPYPRSYYKCTTPGCG VRKHVERAATDPKAVVTTYEGKHNHDLP
AtWRKY6	At1g62300	SEAPMISDGCQWRKYGQKMAKGNPCPRAYYRCTMATGC PVRKQVQRCAEDRSILITTYEGNHNHPLP
AtWRKY7	At4g24240	KMADIPSDEFSWRKYGQKPIKGSPHPRGYYKCSSVRGCPA RKHVERALDDAMMLIVTYEGDHNHALV
AtWRKY8	At5g46350	TEVDHLEDGYRWRKYGQKAVKNSPYPRSYYRCTTQKCN VKKRVERSYQDPTVVITTYESQHNHPIP
AtWRKY9	At1g68150	CETATMNDGCQWRKYGQKTAKGNPCPRAYYRCTVAPGC PVRKQVQRCLEDMSILITTYEGTHNHPLP
AtWRKY10	At1g55600	SDEDNPNDGYRWRKYGQKVVKGNPNPRSYFKCTNIECRV KKHVERGADNIKLVVTTYDGIHNHPSP
AtWRKY11	At4g31550	KIADIPPDEYSWRKYGQKPIKGSPHPRGYYKCSTFRGCPA RKHVERALDDPAMLIVTYEGEHRHNQS
AtWRKY12	At2g44745	SDVDVLDDGYKWRKYGQKVVKNSLHPRSYYRCTHNNCR VKKRVERLSEDCRMVITTYEGRHNHIPS
AtWRKY13	At4g39410	SEVDVLDDGYRWRKYGQKVVKNTQHPRSYYRCTQDKCR VKKRVERLADDPRMVITTYEGRHLHSPS
AtWRKY14	At1g30650	SGEVVPSDLWAWRKYGQKPIKGSPFPRGYYRCSSSKGCS ARKQVERSRTDPNMLVITYTSEHNHPWP
AtWRKY15	At2g23320	KMSDVPPDDYSWRKYGQKPIKGSPHPRGYYKCSSVRGCP ARKHVERAADDSSMLIVTYEGDHNHSLS
AtWRKY16	At5g45050	DRGSRSSDLWVWRKYGQKPIKSSPYPRSYYRCASSKGCF ARKQVERSRTDPNVSVITYISEHNHPFP

AtWRKY17	At2g24570	KIADIPPDEYSWRKYGQKPIKGSPHPRGYYKCSTFRGCPA RKHVERALDDSTMLIVTYEGEHRHHQS
AtWRKY18	At4g31800	DTSLTVKDGFQWRKYGQKVTRDNPSPRAYFRCSFAPSCP VKKKVQRSAEDPSLLVATYEGTHNHLGP
AtWRKY19N	At4g12020	NVDKQVNDGYNWQKYGQKKVKGSKFPLSYYKCTYLGC PSKRKVERSLDGQVAEIVYKDRHNHEPP
AtWRKY19C	At4g12020	SEVDNLDDGYRWRKYGQKVVKGNPYPRFSSSKDYDVVI RYGRADISNEDFISHLRASLCRRGISVYEKFNEVDALPKCR VLIIVLTSTYVPSNLLNILEHQHTED
AtWRKY20N	At4g26640	TPSILADDGYNWRKYGQKHVKGSEFPRSYYKCTHPNCEV KKLFERSHDGQITDIIYKGTHDHPKP
AtWRKY20C	At4g26640	SEVDILDDGYRWRKYGQKVVRGNPNPRSYYKCTAHGCP VRKHVERASHDPKAVITTYEGKHDHDVP
AtWRKY21	At2g30590	KVADIPPDDYSWRKYGQKPIKGSPYPRGYYKCSSMRGCP ARKHVERCLEDPAMLIVTYEAEHNHPKL
AtWRKY22	At4g01250	AAEALNSDVWAWRKYGQKPIKGSPYPRGYYRCSTSKGCL ARKQVERNRSDPKMFIVTYTAEHNHPAP
AtWRKY23	At2g47260	SEVDHLEDGYRWRKYGQKAVKNSPFPRSYYRCTTASCNV KKRVERSFRDPSTVVTTYEGQHTHISP
AtWRKY24	At5g41570	SDDDVLDDGYRWRKYGQKSVKHNAHPRSYYRCTYHTCN VKKQVQRLAKDPNVVVTTYEGVHNHPCE
AtWRKY25N	At2g30250	MVSRNSNDGYGWRKYGQKQVKKSENPRSYFKCTYPDCV SKKIVETASDGQITEIIYKGGHNHPKP
AtWRKY25C	At2g30250	SDIDVLIDGFRWRKYGQKVVKGNTNPRSYYKCTFQGCGV KKQVERSAADERAVLTTYEGRHNHDIP
AtWRKY26N	At5g07100	SSNKTSDDGYNWRKYGQKQVKGSENPRSYFKCTYPNCLT KKKVETSLVKGQMIEIVYKGSHNHPKP
AtWRKY26C	At5g07100	SDIDILDDGYRWRKYGQKVVKGNPNPRSYYKCTFTGCFV RKHVERAFQDPKSVITTYEGKHKHQIP
AtWRKY27	At5g52830	TQENLSSDLWAWRKYGQKPIKGSPYPRNYYRCSSSKGCL ARKQVERSNLDPNIFIVTYTGEHTHPRP
AtWRKY28	At4g18170	SEVDHLEDGYRWRKYGQKAVKNSPYPRSYYRCTTQKCN VKKRVERSFQDPTVVITTYEGQHNHPIP
AtWRKY29	At4g23550	KEENLLSDAWAWRKYGQKPIKGSPYPRSYYRCSSSKGCL ARKQVERNPQNPEKFTITYTNEHNHELP
AtWRKY30	At5g24110	GVDRTLDDGFSWRKYGQKDILGAKFPRGYYRCTYRKSQ GCEATKQVQRSDENQMLLEISYRGIHSCSQA
AtWRKY31	At4g22070	SEAAMISDGCQWRKYGQKMAKGNPCPRAYYRCTMAGG CPVRKQVQRCAEDRSILITTYEGNHNHPLP
AtWRKY32N	At4g30935	VPRTPARDGYNWRKYGQKQVKSPKGSRSYYRCTYTECC AKKIECSNDSGNVVEIVNKGLHTHEPP
AtWRKY32C	At4g30935	GDVGICGDGYRWRKYGQKMVKGNPHPRNYYRCTSAGCP VRKHIETAVENTKAVIITYKGVHNHDMP

AtWRKY33N	At2g38470	REQRKGEDGYNWRKYGQKQVKGSENPRSYYKCTFPNCP TKKKVERSLEGQITEIVYKGSHNHPKP
AtWRKY33C	At2g38470	SDIDILDDGYRWRKYGQKVVKGNPNPRSYYKCTTIGCPV RKHVERASHDMRAVITTYEGKHNHDVP
AtWRKY34N	At4g26440	ACCAPADDGYNWRKYGQKLVKGSEYPRSYYKCTHPNCE AKKKVERSREGHIIEIIYTGDHIHSKP
AtWRKY34C	At4g26440	SDIDILDDGYRWRKYGQKVVKGNPNPRSYYKCTANGCTV TKHVERASDDFKSVLTTYIGKHTHVVP
AtWRKY35	At2g34830	SGEVVPSDLWAWRKYGQKPIKGSPYPRGYYRCSSSKGCS ARKQVERSRTDPNMLVITYTSEHNHPWP
AtWRKY36	At1g69810	CEDPSINDGCQWRKYGQKTAKTNPLPRAYYRCSMSSNCP VRKQVQRCGEEETSAFMTTYEGNHDHPLP
AtWRKY38	At5g22570	SPDPIYYDGYLWRKYGQKSIKKSNHQRSYYRCSYNKDHN CEARKHEQKIKDNPPVYRTTYFGHHTCKTE
AtWRKY39	At3g04670	KIADIPPDEYSWRKYGQKPIKGSPHPRGYYKCSSVRGCPA RKHVERCIDETSMLIVTYEGEHNHSRI
AtWRKY40	At1g80840	DTTLVVKDGYQWRKYGQKVTRDNPSPRAYFKCACAPSC SVKKKVQRSVEDQSVLVATYEGEHNHPMP
AtWRKY41	At4g11070	GLEGPHDDIFSWRKYGQKDILGAKFPRSYYRCTFRNTQYC WATKQVQRSDGDPTIFEVTYRGTHTCSQG
AtWRKY42	At4g04450	SEAPMLSDGCQWRKYGQKMAKGNPCPRAYYRCTMAVG CPVRKQVQRCAEDRTILITTYEGNHNHPLP
AtWRKY43	At2g46130	SDADILDDGYRWRKYGQKSVKNSLYPRSYYRCTQHMCN VKKQVQRLSKETSIVETTYEGIHNHPCE
AtWRKY44N	At2g37260	TGDRSSVDGYNWRKYGQKQVKGSECPRSYYKCTHPKCP VKKKVERSVEGQVSEIVYQGEHNHSKP
AtWRKY44C	At2g37260	VESDSLEDGFRWRKYGQKVVGGNAYPRSYYRCTSANCR ARKHVERASDDPRAFITTYEGKHNHHLL
AtWRKY45	At3g01970	SQVDILDDGYRWRKYGQKAVKNNPFPRSYYKCTEEGCR VKKQVQRQWGDEGVVVTTYQGVHTHAVD
AtWRKY46	At2g46400	QENGSIDDGHCWRKYGQKEIHGSKNPRAYYRCTHRFTQD CLAVKQVQKSDTDPSLFEVKYLGNHTCNNI
AtWRKY47	At4g01720	SDATTVNDGCQWRKYGQKMAKGNPCPRAYYRCTMAVG CPVRKQVQRCAEDTTILTTTYEGNHNHPLP
AtWRKY48	At5g49520	SDIDNLDDGYRWRKYGQKAVKNSPYPRSYYRCTTVGCG VKKRVERSSDDPSIVMTTYEGQHTHPFP
AtWRKY49	At5g43290	NSNGMCDDGYKWRKYGQKSIKNSPNPRSYYKCTNPICNA KKQVERSIDESNTYIITYEGFHFHYTY
AtWRKY50	At5g26170	SEVEVLDDGFKWRKYGKKMVKNSPHPRNYYKCSVDGCP VKKRVERDRDDPSFVITTYEGSHNHSSM
AtWRKY51	At5g64810	SKIDVMDDGFKWRKYGKKSVKNNINKRNYYKCSSEGCS VKKRVERDGDDAAYVITTYEGVHNHESL
AtWRKY52	At5g45260	IPAIDEGDLWTWRKYGQKDILGSRFPRGYYRCAYKFTHG CKATKQVQRSETDSNMLAITYLSEHNHPRP

AtWRKY53	At4g23810	GLEGPQDDVFSWRKYGQKDILGAKFPRSYYRCTHRSTQN CWATKQVQRSDGDATVFEVTYRGTHTCSQA
AtWRKY54	At2g40750	VEAKSSEDRYAWRKYGQKEILNTTFPRSYFRCTHKPTQGC KATKQVQKQDQDSEMFQITYIGYHTCTAN
AtWRKY55	At2g40740	NTDLPPDDNHTWRKYGQKEILGSRFPRAYYRCTHQKLYN CPAKKQVQRLNDDPFTFRVTYRGSHTCYNS
AtWRKY56	At1g64000	SDDDVLDDGYRWRKYGQKSVKNNAHPRSYYRCTYHTCN VKKQVQRLAKDPNVVVTTYEGVHNHPCE
AtWRKY57	At1g69310	SDVDNLEDGYRWRKYGQKAVKNSPFPRSYYRCTNSRCT VKKRVERSSDDPSIVITTYEGQHCHQTI
AtWRKY58N	At3g01080	NVDKPADDGYNWRKYGQKPIKGCEYPRSYYKCTHVNCP VKKKVERSSDGQITQIIYKGQHDHERP
AtWRKY58C	At3g01080	SEVDLLDDGYRWRKYGQKVVKGNPHPRSYYKCTTPNCT VRKHVERASTDAKAVITTYEGKHNHDVP
AtWRKY59	At2g21900	DEKVALDDGYKWRKYGKKPITGSPFPRHYHKCSSPDCNV KKKIERDTNNPDYILTTYEGRHNHPSP
AtWRKY60	At2g25000	DTSLTVKDGYQWRKYGQKITRDNPSPRAYFRCSFSPSCLV KKKVQRSAEDPSFLVATYEGTHNHTGP
AtWRKY61	At1g18860	CETPTMNDGCQWRKYGQKIAKGNPCPRAYYRCTIAASCP VRKQVQRCSEDMSILISTYEGTHNHPLP
AtWRKY62	At5g01900	SSTPIYHDGFLWRKYGQKQIKESEYQRSYYKCAYTKDQN CEAKKQVQKIQHNPPLYSTTYFGQHICQLHQA
AtWRKY63	At1g66600	SPNPRLDDGFTWRKYGQKTIKTSLYQRCYYRCAYAKDQN CYATKRVQMIQDSPPVYRTTYLGQHTCKAF
AtWRKY64	At1g66560	SPTPRPDDGFTWRKYGQKTIKTSPYQRCYYRCTYAKDQN CNARKRVQMIQDNPPVYRTTYLGKHVCKAV
AtWRKY65	At1g29280	GDTTPPSDSWAWRKYGQKPIKGSPYPRGYYRCSSTKGCP ARKQVERSRDDPTMILITYTSEHNHPWP
AtWRKY66	At1g80590	SPTPAHIDGFIWRKYGQKTIKTSPHQRWYYRCAYAKDQN CDATKRVQKIQDNPPVYRNTYVGQHACEAP
AtWRKY67	At1g66550	SRTMCPNDGFTWRKYGQKTIKASAHKRCYYRCTYAKDQ NCNATKRVQKIKDNPPVYRTTYLGKHVCKAF
AtWRKY68	At3g62340	SEVLHLDDGYKWRKYGQKPVKDSPFPRNYYRCTTTWCD VKKRVERSFSDPSSVITTYEGQHTHPRP
AtWRKY69	At3g58710	GEVYPPSDSWAWRKYGQKPIKGSPYPRGYYRCSSSKGCP ARKQVERSRVDPSKLMITYACDHNHPFP
AtWRKY70	At3g56400	IESTILEDAFSWRKYGQKEILNAKFPRSYFRCTHKYTQGCK ATKQVQKVELEPKMFSITYIGNHTCNTN
AtWRKY71	At1g29860	SEIDHLEDGYRWRKYGQKAVKNSPYPRSYYRCTTQKCNV KKRVERSFQDPSIVITTYEGKHNHPIP
AtWRKY72	At5g15130	CDTPTMNDGCQWRKYGQKIAKGNPCPRAYYRCTVAPGC PVRKQVQRCADDMSILITTYEGTHSHSLP
AtWRKY74	At5g28650	KIADIPPDEYSWRKYGQKPIKGSPHPRGYYKCSSVRGCPA RKHVERCVEETSMLIVTYEGEHNHSRI

AtWRKY75	At5g13080	SQVDILDDGYRWRKYGQKAVKNNKFPRSYYRCTYGGCN VKKQVQRLTVDQEVVVTTYEGVHSHPIE
BdWRKY1	Bradi2g16150.1	ISDGCQWRKYGQKMAKGNPCPRAYYRCTMAAGCPVRK QVQRCAEDRTVLITTYEGNHNHPL
BdWRKY2	Bradi2g05510.1	MEDGFRWRKYGKKAVKSSPNLRNYYRCSAPGCGVKKRV ERDRHDPAYVITTYHGVHNHPT
BdWRKY3	Bradi2g62130.1	ADDGYKWRKYGQKSIKNSPNPRSYYRCTNPRCNAKKQV ERSTEEPDTLLVTYEGLHLHYT
BdWRKY4	Bradi2g11170.1	ISDGCQWRKYGQKMAKGNPCPRAYYRCTMATGCPVRKQ VQRCAEDKTVLITTYEGSHNHQL
BdWRKY5	Bradi2g08620.1	IADGCQWRKYGQKMAKGNPCPRAYYRCTMATGCPVRK QVQRCAEDRTILITTYEGTHNHPL
BdWRKY6	Bradi3g18580.1	PGDEFSWRKYGQKPIKGSPYPRGYYKCSTVKGCPARKHV ERATDDPAMLVVTYEGDHRHGA
BdWRKY7N	Bradi4g01950.1	LEDGYNWRKYGQKQVKGSEDPRSYYKCTHAGCSMKKK VERSLADGRVTQIVYKGAHDHPK
BdWRKY7C	Bradi4g01950.1	LDDGFRWRKYGQKVVKGNPNPRSYYKCTTPGCPVRKHV ERASHDARAVITTYEGKHNHDV
BdWRKY8	Bradi2g53510.1	NSDGYMWRKYGQKRIMKTRFPRCYYRCSHHRERGCPAT KQVQEQQHGDGADHPKTYLVIYVHEHTCRT
BdWRKY9	Bradi2g53500.1	YKDGSQWRKYGQKNIRNRIFARYYYKCMYSHERGCRAK KQVQQQDNSSDHRPMFLITYVNEHTCQQ
BdWRKY10	Bradi4g44370.1	HYDGHQWRKYGQKVINNAKHPRSYYRCTYGQEQGCKAT KTVQQKDDNGAGAVYEDDQVMFAVVYYGQHTCKP
BdWRKY11	Bradi2g30695.1	LDDGQAWRKYGQKYIHNSKHPRAYFRCTHKYDQQCAAQ RQVQRCEDDDDDKDTFRVTYIGVHTCRD
BdWRKY12	Bradi3g57710.1	MNDGCQWRKYGQKVAKGNPCPRAYYRCTVAPGCPVRK QVQRCQEDMSILITTYEGTHNHPL
BdWRKY13	Bradi3g34567.1	PSDLWAWRKYGQKPIKGSPYPRGYYRCSSSKGCPARKQV ERSRTDPNMLVITYTSDHNHPW
BdWRKY14	Bradi4g30360.1	VKDGYQWRKYGQKVTRDNPSPRAYFRCAFAPSCPIKKKV QRSAENSSVLEATYEGEHNHPQ
BdWRKY15	Bradi4g44350.1	HYDGHQWRKYGQKNINGMQHSRSYYRCTYKERNCSATK TVQEQDHNRSSFSYGDETVKYTVVYYGHHTCNG
BdWRKY16	Bradi2g45900.1	LEDGYRWRKYGQKAVKNSPYPRSYYRCTTQKCVVKKRV ERSFQDPAVVITTYEGKHTHPI
BdWRKY17	Bradi2g30800.1	PDDGYTWRKYGQKDILGSRYPRSYYRCTHKNYYGCEAK KKVQRLDEDPFTYEVTYCGNHSCLT
BdWRKY18N	Bradi1g02327.1	SYDGYNWRKYGQKQVKGSEFPRSYYKCTYPTCPVKRKV ETTLDGQIAEIVYNGEHNHPK
BdWRKY18C	Bradi1g02327.1	SEDAFRWRKYGQKAVNGNLFPRSYYRCSTARCNARKFVE RSSDNSLCILIA
BdWRKY19	Bradi4g28280.1	GDDGHSWRKYGQKEILGAKHPRGYYRCTHRKTLGCAAT KQVQRSDEDPTLFDVIYHGDHTCLL

BdWRKY20	Bradi1g63220.1	LVDGHVWRKYGQKEIQNSPHPRSYYRCTHKSDQGCNAK RQVQACEADPSKYAVTYYGEHTCSD
BdWRKY21	Bradi3g09810.1	SSDVWAWRKYGQKPIKGSPYPRGYYRCSSSKGCPARKQV ERSRADPNTFILTFTGEHNHAA
BdWRKY22	Bradi1g48770.1	PDDGFSWRKYGQKDILGAKFPRGYYRCTYRTAQGCPATR QVQRSDADLAVFDVTYQGAHTCHQ
BdWRKY23	Bradi1g08106.1	LEDGYRWRKYGQKAVKNSPFPRSYYRCTNSKCTVKKRV ERSSDDPSVVITTYEGQHCHHT
BdWRKY24	Bradi2g49020.1	LDDGYRWRKYGQKAVKNSSNPRSYYRCTHPTCNMKKQV QRLAKDTDIVVTTYEGTHNHPC
BdWRKY25	Bradi2g53520.1	TEDGFAWRKYGQKDINGCRHPRLYYRCAYRGEGCVATR RVQRSRDEPAAYAVAYYGEHTCGQ
BdWRKY26	Bradi4g19060.1	LDDGYRWRKYGQKAVKNNNFPRSYYRCTHQGCNVKKQ VQRLSRDEGVVVTTYEGTHTHPI
BdWRKY27N	Bradi1g07970.1	GKDGYNWRKYGQKQLKDAESPRSYYKCTREACPVKKIV ERSFDGCIKEITYKGRHTHPR
BdWRKY27C	Bradi1g07970.1	LDDGYRWRKYGQKVVKGNPRPRSYYKCTAENCNVRKQI ERASSNPSCVLTTYTGRHSHHP
BdWRKY28	Bradi1g17660.1	PEDGQSWRKYGQKFIHKSTNPRSYYRCTHKHDQGCKATK QVQKSESNPSEFVISYFGEHTCKD
BdWRKY29	Bradi4g44360.1	HYDGHQWRKYGQKNINGMQHPRSYYRCTYKERSCSATK TVQKQDHNGSSFSYGDEAVNYTVMYYGNHTCNG
BdWRKY30	Bradi2g15360.1	LDDGYRWRKYGQKAVKNSSYPRSYYRCTAARCGVKKQ VERSQQDPATVITTYEGQHQHPS
BdWRKY31	Bradi1g14300.1	PPDEYSWRKYGQKPIKGSPHPRGYYKCSSVRGCPARKHV ERCVDDPSMLIVTYEGEHNHTR
BdWRKY32N	Bradi5g13090.1	AEDGYSWRKYGQKQVKHSEYPRSYYKCTHQSCQVKKKV ERSHEGHVTEIIYKGTHNHPK
BdWRKY32C	Bradi5g13090.1	LDDGYRWRKYGQKVVKGNPNPRSYYKCTHPGCSVRKHV ERASHDLKSVITTYEGKHNHEV
BdWRKY33	Bradi5g17395.1	LDDGYKWRKYGQKVVKNSLHPRSYYRCTHSNCRVKKRV ERLSEDCRMVITTYEGRHTHTP
BdWRKY34	Bradi5g04817.1	PADDFSWRKYGQKPIKGSPYPRGYYKCSTVRGCPARKHV ERDPSEPSMLIVTYEGDHRHAP
BdWRKY35	Bradi5g20700.1	PADEFSWRKYGQKPIKGSPYPRGYYKCSTVRGCPARKHV ERATDDPAMLVVTYEGEHRHSP
BdWRKY36	Bradi5g20290.1	PSDLWAWRKYGQKPIKGSPHPRGYYRCSSSKGCSARKQV ERSRTDPNMLVITYTSEHNHPW
BdWRKY37N	Bradi3g39340.1	AEDGYNWRKYGQKQVKNSDHPRSYYKCSHPNCPVKKKV ERCQDGHITEIVYKGSHNHPL
BdWRKY37C	Bradi3g39340.1	LDDGYRWRKYGQKVVKGNPNPRSYYRCTHPGCSVRKHV ERASNDPKSVITTYEGKHDHEV
BdWRKY38N	Bradi3g19640.1	VGDGFNWRKYGQKQVKSSDNSRSYYRCTNSSCLAKKKV EHYPDGRVIEIIYRGTHSHEP

BdWRKY38C	Bradi3g19640.1	MSDGYRWRKYGQKIVKGNPNPRSYYRCTHDGCPVRKHV EKAADDINNMVVTYEGKHNHDQ
BdWRKY39	Bradi3g06070.1	VKDGYQWRKYGQKVTKDNPCPRAYFRCSFAPACPVKKK VQRSAEDRTVLVATYEGEHNHGQ
BdWRKY40	Bradi3g34850.1	GEDGHSWRKYGQKDILGAKHPRGYYRCTHRNSQGCAAT KQVQRADHDPALFDVVYHGEHTCRA
BdWRKY41	Bradi3g50360.1	LDDGYKWRKYGQKVVKNSLHPRSYFRCTQSNCRVKKRV ERLSTDCRMVITTYEGRHTHSP
BdWRKY42	Bradi3g52420.1	PSDLWAWRKYGQKPIKGSPYPRGYYRCSSSKGCPARKQV ERSRTDPNTLVITYTSEHNHPW
BdWRKY43	Bradi4g25717.1	YDDGHQWRKYGEKKLSNSNFPRFYYRCTYKNDMKCPAT KQVQQKDTSDPPLFSVTYFNHHTCNT
BdWRKY44	Bradi2g22230.1	ENDGFHWRKYGEKKILNAAFPRLYYRCGYSDEHKCPAKK YVQQQDNGDPPLFMVTLINDHTCDA
BdWRKY45N	Bradi2g53760.1	SDDGYNWRKYGQKQVKGSENPRSYYKCTFPSCPTKKKV ETSLEGQITEIVYKGTHNHAK
BdWRKY45C	Bradi2g53760.1	LDDGYRWRKYGQKVVKGNPNPRSYYKCTTVGCPVRKHV ERASQDLRAVITTYEGKHNHDV
BdWRKY46N	Bradi2g22440.1	SDDGYNWRKYGQKQMKGSENPRSYYKCSAPGCPTKKKV EQAPDGHVTEIVYKGTHNHPK
BdWRKY46C	Bradi2g22440.1	LDDGYRWRKYGQKVVKGNPNPRSYYKCTMAGCPVRKH VERASQDLRAVVTTYEGKHNHDV
BdWRKY47N	Bradi2g00280.1	LEDGYKWRKYGQKQVKGSENPRSYYKCTYSNCSMKKKV ERSLADGRITQIVYKGAHHHPK
BdWRKY47C	Bradi2g00280.1	LDDGFRWRKYGQKVVKGNPNPRSYYKCTTVACPVRKHV ERASHDNRAVITTYEGKHNHDV
BdWRKY48	Bradi2g05500.1	MNDGCQWRKYGQKISKGNPCPRAYYRCTVAPSCPVRKQ VQRCADDMSILITTYEGAHTHPL
BdWRKY49	Bradi2g05234.1	LDDGYKWRKYGQKVVKNTHHPRSYYRCTQDKCRVKKR VERLAEDPRMVITTYEGRHVHSP
BdWRKY50	Bradi2g33540.1	LEDGFKWRKYGKKAVKNSPNPRNYYRCSAERCGVKKRV ERDRDDPRFVVTTYDGVHNHAT
BdWRKY51	Bradi2g15877.1	LDDGLSWRKYGQKDILGAKYPRAYFRCTHRNTQGCQAT KQVQRDAGDPLIFDVVYHGDHTCAQ
BdWRKY52	* LOC100843345	MDDGYRWRKYGKKMVKNSPNPRNYYRCSSEGCRVKKR VERERDDARFVITTYHGVHDHPA
BdWRKY53	Bradi2g44090.1	LEDGYRWRKYGQKAVKNSPYPRSYYRCTTPKCGVKKRV ERSYQDPSTVITTYEGQHTHHS
BdWRKY54	Bradi2g19070.1	LDDGYRWRKYGQKAVKNSAFPRSYYRCTHHTCNVKKQV QRLAKDTAVVVTTYEGVHNHPC
BdWRKY55	Bradi2g45480.1	LEDRFSWRKYGQKDILGAKYPRAYFRCTHRHTQSCSASK QVQRTDGDPLLFDVVYHGNHTCAQ
BdWRKY56	Bradi2g18530.1	LDDGYKWRKYGKKSVKNSPNPRNYYRCSTEGCSVKKRV ERDRDDPSYVVTTYEGTHSHVS

BdWRKY57	Bradi2g54720.1	PTDGYKWRKYGQKSIKNNPHPRSYYKCTSSRCSAKKHVE KSTHDPEMFTVTYEGLHLHGP
BdWRKY58	Bradi2g48090.1	LDDGYKWRKYGKKSVKNSPNPRNYYRCSTEGCNVKKRV ERDRDDADYVLTMYEGIHNHAS
BdWRKY59N	Bradi4g45290.1	SYDGYNWRKYGQKQVKGSEFPRSYYKCTYPTCPVKRKV ETTLDGQIAEIVYNGEHNHPK
BdWRKY59C	Bradi4g45290.1	SEDAFRWRKYGQKAVNGNLFPRSYYRCSTARCNARKFVE RSSDNSLVTTYEGRHNHIA
BdWRKY60N	Bradi4g33370.1	DEDGYNWKKYGPKQVKSTEYPRSYFKCTHPNCPVKKKV ERSQVGQITEIIYKGTHNHPL
BdWRKY60C	Bradi4g33370.1	LDDGYRWRKYGQKVVKGNPNPRSYYKCTHPGCSVRKHV ERASHDLKSVITTYEGKHNHEV
BdWRKY61N	Bradi4g06690.1	ADDGYNWRKYGQKVVKGSDCPRSYYKCTHPSCPVKKKV EHAEDGQISEIIYKGKHNHQR
BdWRKY61C	Bradi4g06690.1	LDDGYRWRKYGQKVVKGNPHPRSYYKCTFAGCNVRKHI ERASSDPKAVITTYEGKHNHEP
BdWRKY62N	Bradi4g09890.1	TDDGYNWRKYGQTQVKGSENPRSYYKCAFPSCPAKKKV ERSLDNQIIEILYKGRHNHPK
BdWRKY62C	Bradi4g09890.1	ADDGYNWRKYGQTQVKGSENLRSYYKCTFPNCSTKKKV ERSLANHIIEILYKGRHNHPK
BdWRKY63	Bradi4g02680.1	PPDEYSWRKYGQKPIKGSPHPRGYYKCSSVRGCPARKHV ERCVDEPAMLAVTYEGEHNHNR
BdWRKY64N	Bradi1g23340.1	AEDGYNWRKYGQKHVKGSENPRSYYKCTHPNCEVKKLL ERAADGQITEVVYKGRHNHPK
BdWRKY64C	Bradi1g23340.1	LDDGYRWRKYGQKVVKGNPNPRSYYKCTSTGCPVRKHV ERASHDPKSVITTYEGKHNHEV
BdWRKY65N	Bradi1g47690.1	SYDGYNWRKYGQKQVKGSEFPRSYYKCTYPTCPVKRKV ETTLDGQIAEIVYNGEHNHPK
BdWRKY65C	Bradi1g47690.1	SEDAFRWRKYGQKAVNGNLFPIYGKSRLNVHMKKCMQE LLCGCSGKPIKDNSRSTRCGCAA
BdWRKY66N	Bradi1g16120.1	ADDGYNWRKYGQKAVKGGEYPRSYYKCTQAGCPVKKK VERSACGEITQIIYRGQHNHQR
BdWRKY66C	Bradi1g16120.1	LDDGYRWRKYGQKVVKGNPHPRSYYKCTFQGCDVKKHI ERCSQDSTDVITTYEGKHSHDV
BdWRKY67N	Bradi1g22680.1	ADDGYNWRKYGQKAVKGGRYPRSYYKCTLNCPVRKNV EHSEDGKIIKIIYRGQHSHER
BdWRKY67C	Bradi1g22680.1	LDDGYRWRKYGQKVVRGNPHPRSYYKCTYQGCDVKKHI ERSSQEPHAVITTYEGKHVHDV
BdWRKY68	Bradi1g30870.1	VKDGYQWRKYGQKVTKDNPCPRAYFRCSFAPACPVKKK VQRSADDKALLVATYEGDHNHAQ
BdWRKY69	* LOC100834454	EADPWAWRKYGQKTVKGSPYTRSYYRCSTAKECGARKI MELCPTDPDTLILTYTGADHNHPP
BdWRKY70	Bradi1g51030.1	MNDGCQWRKYGQKISKGNPCPRAYYRCTVAAGCPVRKQ VQRCAEDMSILISTYEGRHNHPL

BdWRKY71	Bradi1g59180.1	LEDGYRWRKYGQKAVKNSPFPRSYYRCTNSKCTVKKRV ERSSNDPSIVITTYEGQHCHHT
BdWRKY72	Bradi1g13207.1	PEDGYEWKKYGQKFIKNIQRIRSYFRCRDKRCGAKKKVE WQPGDPSLRVVYEGAHQHGS
BdWRKY73	* LOC100845846	PSDLWAWRKYGQKPIKGSPYPRGYYRCSSSKGCMARKQ VERSRSDPNMLVITYTAEHNHPW
BdWRKY74	Bradi1g09170.1	PGDEFSWRKYGQKPIKGSPHPRGYYKCSSVRGCPARKHV ERCVDDPAMLIVTYEGDHNHNR
BdWRKY75	* LOC100837754	PGDEFSWRKYGQKRIKGFPHPRQQNRIMKKEKLNERISKL AGGVAVIQAAVEEGNVVRGGCAL
BdWRKY76	Bradi2g16357.1	LEDGYRWRKYGQKAVKNSPFPRSYYRCTAQKCPVKKRV ERSFQDAAVVITTYEGKHTHPI
BdWRKY77	Bradi2g49906.1	PPDSWAWRKYGQKPIKGSPYPRGYYRCSSSKGCPARKQV ERSRTDPTVLLVTYSFDHNHPW
BdWRKY78	Bradi1g63910.1	HNDGHQWRKYGEKKINNCNFPRYYYRCTYKDNMNCPAT KQIQQKDHSDPPLYQVTYYNEHSCNS
BdWRKY79	Bradi2g44270.1	VEDGQSWRKYGQKDIQNSEHPKSYFRCTHKYDQKCAAL RQVQRCDQDPESFVVTYIGQHTCQD
BdWRKY80	Bradi2g48907.1	PTDEWAWRKYGQKPIKGSPFPSRAYYRCSSSKGCPARKQ VERSQADPAMVLVTYSYEHNHST
BdWRKY81	Bradi2g15405.1	ADDLWAWRKYGQKPIKGSPYPRGYYKCSSLKACAARKL VERSPDKPEVLIVTYIADHCHAV
BdWRKY82	Bradi2g44035.1	SSDLWAWRKYGQKPIKGSPYPRGYYKCSSMKGCMARKM VERSPAKPGVLVITYMAEHCHPV
BdWRKY83	Bradi2g22240.1	VEDGYIWRKYGQKEILNSSHPRLYFRCSYKHDSGCPATRQ VQHSDHDPSLYVITYFGHHTCCV
BdWRKY84	Bradi2g53480.1	YHDGFQWRKYGQKMIRGNIFPRCYYRCTYHQDHGCPAS KHVEQSNSEDPPLFRVIYTNDHTCSG
BdWRKY85	Bradi2g53495.1	HFDGHLWRKYGQKNIKDSAFPRLYYRCSYREDKRCLASK LVQQENCDDPPLFKVTYTYEHSCNT
OsWRKY1	LOC_Os10g42850.1	PSDLWAWRKYGQKPIKGSPYPRGYYRCSSSKGCSARKQV ERSRADPTMLVVTYTSDHNHPW
OsWRKY3	LOC_Os03g55080.1	LEDGYRWRKYGQKAVKNSPFPRSYYRCTNSKCTVKKRV ERSSDDPSVVITTYEGQHCHHT
OsWRKY4	LOC_Os06g44010.1	VKDGYQWRKYGQKVTKDNPCPRAYFRCSFAPACPVKKK VQRSADDNTVLVATYEGEHNHAQ
OsWRKY5	LOC_Os06g05380.1	MNDGCQWRKYGQKIAKGNPCPRAYYRCTVAAGCPVRK QVQRCADDMSILITTYEGTHNHPL
OsWRKY6	LOC_Os06g30860.1	APDLWAWRKYGQKPIKGSPYPRGYYRCSSNKNCAARKQ VERCRFDPSFLLLTYTGAHSGHDV
OsWRKY7	LOC_Os11g02470.1	HEDGFQWRKYGEKKIQGTHFTRSYFRCTYRDDRGCQATK QIQQKDKNDPPMFQVTYSNEHTCTT
OsWRKY8	LOC_Os11g02480.1	YDDGHQWRKYGEKKLSNSNFPRFYYRCTYKNDMKCPAT KQVQQKDTNDPPLFSVTYFNHHTCNS

OsWRKY9	LOC_Os11g02520.1	HNDGHQWRKYGQKWISRAKHSRSYYRCANSKVQGCPAT KTVQQMDSSGNGTSKLFNVDYYGQHTCRG
OsWRKY10	LOC_Os11g02530.1	HYDGHQWRKYGQKHINNSKHPRSYYRCTYRQEEKCKAT KTVQQREDLHHANSYNGDHPIMYTVVYYGQHTCCK
OsWRKY11	LOC_Os11g02540.1	DFDGYQWRKYGQKQIEGALYPRSYYRCTNSTNQGCLAK KTVQRNGGGGAAGYTVAYISEHTCKS
OsWRKY12	LOC_Os02g43560.1	LDDGYKWRKYGQKVVKNSLHPRYICST*
OsWRKY13	LOC_Os09g30400.1	LDDGYRWRKYGQKVVKGNPNPRSYYKCTHQGCSVRKH VERASHDLKSVITTYEGKHNHEV
OsWRKY14	LOC_Os09g25070.1	VKDGYQWRKYGQKVTRDNPYPRAYFRCAFAPSCPVKKK LQRCAEDRSMLVATYEGEHNHAL
OsWRKY15	LOC_Os08g09800.1	EYDGYNWRICGQKVVQGGCHQKFYYECSQANCGAEKSV TRSADGQIKKTVCKGSHNHPL
OsWRKY16	LOC_Os08g09810.1	EYDGYNWRMCGQKLVQGGCHQKFYYECSQANCGAEKS VTRSADGQIKKTVCKGSHNHPR
OsWRKY17	LOC_Os02g53100.1	MNDGCQWRKYGQKVAKGNPCPRAYYRCTVAPGCPVRK QVQRCLEDMSILVTTYEGTHNHPL
OsWRKY18	LOC_Os02g08440.1	VKDGYQWRKYGQKVTKDNPCPRAYFRCSFAPACPVKKK VQRSAEDNTILVATYEGEHNHGQ
OsWRKY19	LOC_Os03g21710.1	LEDGHVWRKYGQKDIQNSPYPRSYYRCTHKLDQGCGAR RQTQRCEADPSNYDITYYGEHTCRD
OsWRKY20	LOC_Os01g40260.1	LDDGYRWRKYGKKMVKNSPNPRNYYRCSSEGCRVKKRV ERARDDARFVVTTYDGVHNHPA
OsWRKY22N	LOC_Os01g61080.1	SDDGYNWRKYGQKQVKGSENPRSYYKCTFPNCPTKKKV ERSLDGQITEIVYKGTHNHAK
OsWRKY22C	LOC_Os01g61080.1	LDDGYRWRKYGQKVVKGNPNPRSYYKCTTAGCPVRKHV ERASHDLRAVITTYEGKHNHDV
OsWRKY23	LOC_Os01g43550.1	SSDLWAWRKYGQKPIKGSPYPRGYYKCSSMKGCMARKM VERSPAKPGMLVVTYMAEHCHPV
OsWRKY24	LOC_Os01g43650.1	LEDGYRWRKYGQKAVKNSPYPRSYYRCTTPKCGVKKRV ERSYQDPSTVITTYEGQHTHHS
OsWRKY25	LOC_Os03g45450.1	PEDGYEWKKYGQKFIKNIQKNRSYFRCRDQRCGAKKKVE WHPHDP
OsWRKY26	LOC_Os01g09080.1	MNDGCQWRKYGQKISKGNPCPRAYYRCTVAPNCPVRKQ VQRCADDMSILITTYEGTHSHPL
OsWRKY27	LOC_Os01g09100.1	LDDGFKWRKYGKKAVKSSPNPRNYYRCSAAGCGVKKRV ERDGDDPRYVVTTYDGVHNHAT
OsWRKY28	LOC_Os01g74140.1	ADDGYKWRKYGQKSIKNSPNPRSYYRCTNPRCNAKKQV ERAVDEPDTLIVTYEGLHLHYT
OsWRKY30	LOC_Os04g51560.1	PPDEYSWRKYGQKPIKGSPYPRGYYKCSTVRGCPARKHV ERATDDPAMLVVTYEGEHRHTP
OsWRKY31	LOC_Os01g53260.1	LDDGYRWRKYGQKAVKNSKHPRSYYRCTHHTCNVKKQ VQRLAKDTSIVVTTYEGVHNHPC

OsWRKY32	LOC_Os01g40430.1	VKDGCQWRKYGQKTAKGNPWPRGYYRCTGAPGCPVKK QVQRCNHDTSVLVTTYDGVHNHPI
OsWRKY33	LOC_Os01g53040.1	PTDSWAWRKYGQKPIKGSPFPRAYYRCSSSKGCPARKQV ERSRNDPDTVIVTYSFEHNHSA
OsWRKY34	LOC_Os04g50920.1	PSDLWAWRKYGQKPIKGSPYPRGYYRCSSSKGCSARKQV ERSRTDPNMLVITYTSEHNHPW
OsWRKY35	LOC_Os01g47560.1	LEDGYRWRKYGQKAVKNSPYPRSYYRCTTQKCPVKKRV ERSYQDPAVVITTYEGKHTHPI
OsWRKY36	LOC_Os01g51690.1	LDDGYKWRKYGKKSVKNSPNPRNYYRCSTEGCNVKKRV ERDKNDPRYVVTMYEGIHNHVC
OsWRKY37N	LOC_Os04g39570.1	AEDGYSWRKYGQKQVKHSEYPRSYYKCTHASCAVKKKV ERSHEGHVTEIIYKGTHNHPK
OsWRKY37C	LOC_Os04g39570.1	LDDGYRWRKYGQKVVKGNPNPRSYYKCTHPGCLVRKHV ERASHDLKSVITTYEGKHNHEV
OsWRKY38	LOC_Os01g60490.1	YHDGYQWRKYGQKMIRGNSFPRCYYRCTYHQDHGCPAS KHVEQHNSEDPPLFRVIYTNEHTCGT
OsWRKY40	LOC_Os01g60540.1	YKDGYQWRKYGQKNIQDSNYLRLYFKCTFSRERSCAAK KQVQQRDAGEPPMFLVTYLNEHTCQQ
OsWRKY41	LOC_Os01g60600.1	YDDGYQWRKYGQKKINNTNFPRSYYRCSYHRERRCPAQ KHVQQRDGDDVPALHVVVYTHEHTC
OsWRKY42	LOC_Os04g21950.1	PADDFSWRKYGQKPIKGSPFPRGYYKCSTLRGCPARKHV ERDPTDPSMLIVTYEGEHRHSP
OsWRKY43	LOC_Os05g04640.1	ISDGCQWRKYGQKMAKGNPCPRAYYRCTMASQCPVRKQ VQRCAEDKSILITTYEGTHNHPL
OsWRKY44	LOC_Os05g50610.1	LEDGYRWRKYGQKAVKNSSYPSYYRCTAPRCGVKKRVE RSEQDPSMVITTYEGQHTHPS
OsWRKY45	LOC_Os05g14370.1	HYDGHQWRKYGQKNINNSNHQRSYYRCSYKHEQNCKAT KTVQQLDSAGETIMYTVVYYGQHTCKT
OsWRKY46	LOC_Os05g46020.1	LDDGYKWRKYGKKSVKNSPNPRNYYRCSTEGCNVKKRV ERDKDDPSYVVTTYEGTHNHVS
OsWRKY47N	LOC_Os08g38990.1	AEDGYNWRKYGQKQVKNSEHPRSYYKCTFTNCAVKKVE RSQDGQITEIVYKGSHNHPL
OsWRKY47C	LOC_Os08g38990.1	LDDGYRWRKYGQKVVKGNPNPRSYYKCTHPGCSVRKHV ERSSHDLKSVITTYEGKHNHEV
OsWRKY48N	LOC_Os08g17400.1	VGDGFNWRKYGQKQVKSSENSRSYYRCTNSNCLAKKKV EHCPDGRVVEIIYRGTHNHEP
OsWRKY48C	LOC_Os08g17400.1	TSDGYRWRKYGQKIVKGNPNPRSYYRCTHDGCPVRKHV EKAPDDDNNIVVTYEGKHNHDQ
OsWRKY49	LOC_Os07g02060.1	LEDGYRWRKYGQKAVKNSPFPRSYYRCTNSKCTVKKRV ERSSDDPSVVITTYEGQHSHHT
OsWRKY50	LOC_Os02g26430.1	PADDYSWRKYGQKPIKGSPYPRGYYKCSTVRGCPARKHV ERDPGEPAMLIVTYDGDHRHGE
OsWRKY52N	LOC_Os05g03900.1	PADSWSWRKYGQKPIKGSPHPRYHHLALIPHTYTAPNVL VISQSFACALILYITDNFTR

OsWRKY52C	LOC_Os05g03900.1	DHAVSRGYYKCSSYRGCPARKQVDKCRNDASLLIITYTSDHNHDN
OsWRKY53	LOC_Os08g29660.1	ADDGHSWRKYGQKDILGAKHPRGYYRCTHRNTQGCTATKQVQRTDDDASLFDVVYHGEHTCRP
OsWRKY54	LOC_Os02g16540.1	SADVWAWRKYGQKPIKGSPYPRGYYRCSSSKGCPARKQVERSRSDPNTFILTYTGEHNHSA
OsWRKY56	LOC_Os08g13840.1	PADEYSWRKYGQKPIKGSPYPRGYYRCSTVKGCPARKHVERAADDPATLVVTYEGDHRHSP
OsWRKY57	LOC_Os02g47060.1	PSDLWAWRKYGQKPIKGSPYPRGYYRCSSSKGCSARKQVERSRTDPNMLVITYTSEHNHPW
OsWRKY58	LOC_Os09g25060.1	VKDGYQWRKYGQKVTRDNPSPRAYFRCAFAPSCPVKKKVQRSAEDSSLLVATYEGEHNHPH
OsWRKY59N	LOC_Os07g39480.1	AEDGYNWRKYGQKHVKGSENPRSYYKCTHPNCDVKKLLERSLDGQITEVVYKGRHNHPK
OsWRKY59C	LOC_Os07g39480.1	LDDGYRWRKYGQKVVKGNPNPRSYYKCTNTGCPVRKHVERASHDPKSVITTYEGKHNHEV
OsWRKY60	LOC_Os05g50700.1	STDLWAWRKYGQKPIKGSPYPRGYYKCSSLKACMARKMVERSPEKPGVLVITYIAEHCHAV
OsWRKY61N	LOC_Os05g27730.1	LEDGYNWRKYGQKQVKGSENPRSYYKCTYNGCSMKKKVERSLADGRITQIVYKGAHNHPK
OsWRKY61C	LOC_Os05g27730.1	LDDGFRWRKYGQKVVKGNPNPRSYYKCTTVGCPVRKHVERASHDTRAVITTYEGKHNHDV
OsWRKY62	LOC_Os09g09630.1	DYDGYEWRKYGQKSISKTKHSRSYYRCTNQKGQGCMATKTVQQIENDNSSNSVVKLYNVDYFGKHTCKF
OsWRKY63	LOC_Os05g09020.1	LDDGFKWRKYGKKAVKNSPNPRNYYRCSTEGCNVKKRVERDREDHRYVITTYDGVHNHAS
OsWRKY64	LOC_Os06g06360.1	PDDGFSWRKYGQKDILGAKFPRGYYRCTYRNAQGCPATKQVQRSDADLAVFDVTYQGAHTCHQ
OsWRKY65	LOC_Os10g18099.1	HTDGHLWRKYGEKKIKNSSFPRLYYRCSYRDDRNCMATKVVQQENDADPPLYRVTYIHPHTCNP
OsWRKY66	LOC_Os11g45850.1	EDDGFSWRKYGQKDVEGAMHPTTQSNYFRCAHKMTTGCKARKKVQRTDGDPLMVDVVYKGVHSCAG
OsWRKY67	LOC_Os11g45920.1	ADDGYSWRKYGQKNVLGFSYLRGYYRCATKGCQASKQVQRHDDGLLFDVTYFGEHTCAD
OsWRKY68	LOC_Os01g60640.1	TTDGFIWRKYGQKEINGCKHPRLYYRCAFRGQGCLATRRVQQSQSQDDPAAAFVIAYYGEHTCGG
OsWRKY69N	LOC_Os05g39720.1	SDDGYNWRKYGQKQMKGSENPRSYYKCTFPGCPTKKKVEQSPDGQVTEIVYKGAHSHPK
OsWRKY69C	LOC_Os05g39720.1	LDDGYRWRKYGQKVVKGNPNPRSYYKCTTAGCPVRKHVERASNDLRAVITTYEGKHNHDV
OsWRKY70	LOC_Os05g25700.1	PDDGYTWRKYGQKDILGSRYPRSYYRCTHKNYYGCEAKKKVQRLDDDPFTYEVTYCGNHTCLT
OsWRKY71	LOC_Os05g25770.1	LDDGQAWRKYGQKEIQNSKHPKAYFRCTHKYDQLCTAQRQVQRCDDDPASYRVTYIGEHTCRD

OsWRKY72	LOC_Os03g20550.1	HNDGHQWRKYGEKKINNCNFPRYYYRCTYKDNMNCPAT KQIQQKDYSDPPLYSVTYYNEHTCNS
OsWRKY73	LOC_Os12g01180.1	SYDGYSWRKYGQKQVKGSEFPRSYYKCTHPTCPVKRKVE MTPDGRIAEIVYNGEHNHPK
OsWRKY74	LOC_Os12g02470.1	DFDGYQWRKYGQKQIEGAMYPRSYYRCTNSTNQGCLAK KTVQRNGGGGAAGYTVAYISEHTCKS
OsWRKY75	LOC_Os12g02450.1	HYDGHQWRKYGQKHINNSKHPRSYYRCTYRQEEKCKAT KTVQQREDLHHANSYNGDHPVMYTVVYYGQHTCCK
OsWRKY76	LOC_Os12g02420.1	YDDGHQWRKYGEKKLSNSNFPRFYYRCTYKNDMKCPAT KQVQQKDTNDPPLFSVTYFNHHTCNS
OsWRKY77	LOC_Os12g02400.1	HEDGYQWRKYGEKKIQGTHFTRSYFRCTYRDDRGCQAT KQIQQEDKNDPPMFQVTYSNEHTCTT
OsWRKY78	LOC_Os11g29870.1	LDDGYRWRKYGQKAVKNNKFPRSYYRCTHQGCNVKKQ VQRLSRDETVVVTTYEGTHTHPI
OsWRKY79	LOC_Os07g48260.1	LEDGRQWRKYGQKHIQDSPNNPRSYYRCTHRPDQGCMA TKQVQTSESNSSEFVISYYGEHTCSD
OsWRKY81	LOC_Os03g63810.1	PSDLWAWRKYGQKPIKGSPYPRGYYRCSSSKGCMARKQ VERSRSDPNMLVITYAAEHNHPW
OsWRKY83N	LOC_Os12g32250.1	ADDGYNWRKYGQKVVKGSDCPRSYYKCTHPNCPVKKK VEHAEDGQISEIIYKGKHNHQR
OsWRKY83C	LOC_Os12g32250.1	LDDGYRWRKYGQKVVKGNPHPRSYYKCTYAGCNVRKHI ERASSDPKAVITTYEGKHNHEP
OsWRKY84	LOC_Os01g54600.1	PSDSWAWRKYGQKPIKGSPYPRGYYRCSSSKGCPARKQV ERSRADPTVLLVTYSFEHNHPW
OsWRKY85	LOC_Os05g49100.1	LEDGYRWRKYGQKAVKNSPFPRSYYRCTTQKCPVKKRV ERSYQDAAVVITTYEGKHTHPI
OsWRKY86	LOC_Os05g49210.1	ISDGCQWRKYGQKMAKGNPCPRAYYRCTMAAGCPVRK QVQRCAEDRTVLITTYEGNHNHPL
OsWRKY87	LOC_Os07g27670.1	RDDSYPWRKYGQKDILGARFARSYYRCAQMLGCTARKQ VQQSDDDPSRLEITYIGLHTCGG
OsWRKY88	LOC_Os01g18584.1	ISDGCQWRKYGQKMAKGNPCPRAYYRCTMAIGCPVRKQ VQRCAEDKTVLITTYEGNHNHQL
OsWRKY89	LOC_Os12g40570.1	PPDEYSWRKYGQKPIKGSPHPRGYYKCSSVRGCPARKHV ERCVDDPAMLIVTYEENAEAPS
OsWRKY90	LOC_Os01g08710.1	LDDGYKWRKYGQKVVKNTQHPRSYYRCTQDNCRVKKR VERLAEDPRMVITTYEGRHVHSP
OsWRKY93	LOC_Os05g40080.1	MDDKFLWRKYGQKEIKNSKHPRFYYRCSYKDDHGCTAT KQVQQSETADDDTASPVYIITYFGEHTCRH
OsWRKY94	LOC_Os05g40070.1	ENDGFHWRKYGEKNILNSEFRKLYYRCGYSDERKCQAKK YVQQENNKHPPEFRVTLTNEHTCNT
OsWRKY95	LOC_Os05g40060.1	SEDGFLWRKYGQKEIKNSKHPRLYYRCSYKDDHGCTATK QVQQSEEDPSLYVITYFGDHTCSC
OsWRKY96	LOC_Os01g14440.1	IADGCQWRKYGQKMAKGNPCPRAYYRCTMATGCPVRK QVQRCAEDRSILITTYEGTHNHPL

OsWRKY97	LOC_Os01g46800.1	LDDGFSWRKYGQKDILGAKYPRAYFRCTHRHTQGCHAS KQVQRADGDPLLFDVVYHGDHTCAH
OsWRKY100	LOC_Os09g16510.1	VDDGHSWRKYGQKEILGAKHPRGYYRCTHRHSQGCMAT KQVQRTDEDATVFDVIYHGEHTCVH
OsWRKY101	LOC_Os12g02440.1	HNDGHQWRKYGQKWISRAKHSRSYYRCANSKVQGCPAT KTVQQMDSSGNGTSKLFNVDYYGQHTCRG
OsWRKY102	LOC_Os03g58420.1	PSDNYSWRKYGQKPIKGSPHPRGYYRCSSKKDCPARKHV ERCRSDPAMLLVTYENEHNHAQ
OsWRKY103N	LOC_Os07g40570.1	TDDGYNWRKYGQKAVKGGEYPKSYYKCTHLNCLVRKN VEHSADGRIVQIIYRGQHTHER
OsWRKY103C	LOC_Os07g40570.1	LDDGYRWRKYGQKVVKGNPYPRSYYKCTYLGCDVKKQ VERSVEEPNAVITTYEGKHIHDV
OsWRKY105	LOC_Os01g60520.1	HFDGHLWRKYGQKNIKDSAFPRLYYRCSYREDRQCLASK LVQQENDDDPPLYRVTYTYEHTCNT
OsWRKY107N	LOC_Os03g33012.1	ADDGYNWRKYGQKAVKGGEYPRSYYKCTHLSCPVKKK VERSSDGQITQILYRGQHNHQR
OsWRKY107C	LOC_Os03g33012.1	LDDGYRWRKYGQKVVKGNPHPRSYYKCTYQGCDVKKHI ERSSQDPKAVITTYEGKHSHDV
OsWRKY108	LOC_Os01g62514.1	PADGYKWRKYGQKSIKNNPHPRCATRSIIDPI*
OsWRKY109	LOC_Os03g53050.1	PADEFSWRKYGQKPIKGSPHPRGYYKCSSVRGCPARKHV ERCVDDPSMLIVTYEGDHNHNR
OsWRKY110	LOC_Os05g49620.1	LDDGLSWRKYGQKDILGAKYPRAYFRCTHRHTQGCNAT KQVQRADGDPLLFDVVYLGDHTCGQ
OsWRKY111	LOC_Os04g46060.1	LDDGYKWRKYGQKVVKNSLHPRSYYRCTHNNCRVKKR VERLSEDCRMVITTYEGRHTHTP
OsWRKY112	LOC_Os05g45230.1	LDDGYRWRKYGQKAVKNSDFPSDDELLLFSDVDNTQTA TENL
OsWRKY113	LOC_Os08g09840.1	DDDGYYWRMTGQSTTQGESSPTILSHYQCAQANCVVQK TVAYTADVETFYRGRHNHLR
OsWRKY115	LOC_Os08g09900.1	ADDGFHWRMCGQSTIQGGLCPTVFSYQCALPNCGVRKSI TRSADGQTIETVCKGCHNHPR
OsWRKY119	LOC_Os01g62510.1	EDDGLALMTGANQSLWSYYKCTSSRCSAKKHVEKSTDDP EMLIVTYEGSHHHGP
OsWRKY120N	LOC_Os03g55164.1	AKDGYNWRKYGQKQLKDAESPRSYYKCTRDGCPVKKIV ERSSDGCIKEITYKGRHSHPR
OsWRKY120C	LOC_Os03g55164.1	LDDGYRWRKYGQKVVKGNPRPRSYYKCTADGCNVRKQI ERASADPKCVLTTYTGRHNHDP

* GeneBank Locus ID

Supplementary table 4A. Expression data of the BdWRKY family genes after biotic stress treatment.

	F0968-4h			Guy11-4h			PH14-4h			F0968-12h			Guy11-12h			PH14-12h		
gene name	fold-change	p-value	regulation	fold-change	p-value	regulation	fold-change	p-value	regulation	fold-change	p-value	regulation	fold-change	p-value	regulation	fold-change	p-value	regulation
BdWRKY1	0.2831	0.0014	down	563.9	1E-05	up	148.44	3E-05	up	264.52	6E-05	up	0.2368	0.0034	down	200.89	0.0008	up
BdWRKY2	0.842	0.0059		2.0458	6E-06	up	1.0607	0.1618		0.7602	9E-05		2.3793	0.0003	up	1.8278	0.002	up
BdWRKY3	0.545	0.0098	down	0.3352	5E-05	down	4.7204	0.0179	up	1.5179	0.0127	up	15.526	4E-06	up	0.3366	0.0048	down
BdWRKY4	0.7658	0.1127		1.0578	0.9703		1.2865	0.5265		1.171	0.6015		1.1569	0.1135		0.7142	0.1267	
BdWRKY5	0.5492	0.0087	down	0.8406	0.2389		1.1512	0.9768		0.8717	0.2926		1.2706	0.0381		1.3583	0.1282	
BdWRKY6	1.0257	0.8267		2.0885	0.0028	up	1.5089	0.0332	up	0.4782	0.002	down	1.487	0.111		0.7727	0.0416	
BdWRKY7	1.0906	0.3709		1.3242	0.0621		2.6972	3E-05	up	1.8332	0.0004	up	3.1421	4E-06	up	2.0431	0.0006	up
BdWRKY8	0.5673	0.6222		0.8329	0.6188		0.3943	0.5129		0.596	0.744		0.1922	0.0563	down	2.1374	0.0109	up
BdWRKY9	0.4423	0.0049	down	0.6671	0.1412		5.5165	0.0147	up	299.53	0.0001	up	1.8788	0.0389	up	2.6482	0.0316	up
BdWRKY10	0.0009	4E-05	down	0.203	0.0176	down	0.3042	0.0031	down	0.1112	0.0035	down	0.0095	9E-06	down	0.7645	0.1344	
BdWRKY11	0.3942	4E-07	down	0.367	5E-07	down	0.9351	0.0017		0.4788	9E-08	down	1.3458	0.0012		0.7627	4E-05	
BdWRKY12	2.2709	0.0219	up	0.3103	0.0576		0.0163	0.0007	down	1.6201	0.0449	up	0.0624	0.0088	down	2.9578	0.0227	up
BdWRKY13	0.2843	0.0045	down	0.2064	0.0036	down	0.3803	0.0116	down	0.27	0.0067	down	0.762	0.1318		0.3694	0.0112	down
BdWRKY14	0.6393	0.0099	down	0.9657	0.0601		2.0369	0.0261	up	0.9238	0.047		1.9136	0.0081	up	1.7148	0.0441	up
BdWRKY15	0.7413	0.6521		0.2235	0.0041	down	0.2851	0.0011	down	0.2376	0.0017	down	0.684	0.0281		0.3	0.0015	down
BdWRKY16	4.4115	1E-06	up	1.2832	0.0107		1.2916	0.0208		1.6265	0.0023	up	4.3642	0.0013	up	4.5439	0.0009	up
BdWRKY17	0.4545	0.0075	down	0.6935	0.1706		0.982	0.1879		0.3384	0.0022	down	1.1476	0.8818		0.9161	0.3297	
BdWRKY18	70.825	1E-06	up	136.16	2E-07	up	24.844	3E-05	up	8.2594	5E-06	up	456.64	7E-07	up	393.61	2E-06	up
BdWRKY19	0.5226	0.0011	down	0.7115	0.008		0.5691	0.0009	down	0.192	0.0021	down	0.7111	0.0125		0.6772	0.0058	
BdWRKY20	0.6974	0.0134		1.231	0.1379		3.1621	0.0006	up	1.3975	0.0706		2.2055	0.0021	up	1.9483	0.0019	up
BdWRKY21	1.7061	0.0013	up	1.6707	0.001	up	2.9125	0.0001	up	1.0018	0.4061		2.3764	0.0004	up	2.9009	7E-05	up
BdWRKY22	0.1581	0.0011	down	0.1296	3E-05	down	0.6671	0.0245		0.1516	5E-06	down	1.2385	0.8391		0.3466	0.0177	down
BdWRKY23	0.025	1E-05	down	0.0181	1E-05	down	0.0371	1E-05	down	0.0231	0.0011	down	0.0638	0.3802		0.0366	0.0685	
BdWRKY24	0.2721	0.0005	down	0.3279	0.0005	down	1.0668	0.6862		0.5382	0.0216	down	1.3779	0.334		0.5331	0.0192	down
BdWRKY25	3.2928	0.0039	up	0.162	0.091	down	1.3441	0.7595		1.6011	0.0091	up	2.0996	0.0308	up	4.5041	0.0005	up
BdWRKY26	0.5787	2E-05	down	0.733	0.0062		1.4134	0.0033		0.6458	3E-06	down	1.2394	0.3778		0.9497	0.0041	

BdWRKY27	0.6722	0.2146		0.0832	0.0014	down	0.1991	0.0071	down	0.1809	0.0153	down	0.2039	0.0044	down	0.1967	0.0196	down
BdWRKY28	0.4803	3E-05	down	0.275	3E-06	down	0.4748	0.0003	down	0.1583	1E-06	down	0.671	0.0107		0.447	0.0042	down
BdWRKY29	0.8177	0.7122		0.2644	0.1634		0.3593	2E-05	down	3.233	0.0018	up	1.599	0.022	up	0.2078	0.0259	down
BdWRKY30	8.6772	0.0003	up	9.7718	0.0003	up	11.323	0.0002	up	9.1166	0.0003	up	11.055	0.0002	up	14.554	0.0001	up
BdWRKY31	0.3532	0.0013	down	0.3363	0.0002	down	0.5304	0.0001	down	0.4367	0.0007	down	0.8136	0.0062		0.4031	6E-05	down
BdWRKY32	0.4337	0.1041		0.1233	0.006	down	0.8115	0.2364		0.3057	0.0107	down	0.1783	0.0105	down	0.236	0.0203	down
BdWRKY33	0.517	0.0034	down	0.4475	0.0003	down	0.7648	0.0056		0.5546	0.0008	down	1.2115	0.9015		0.5664	0.0011	down
BdWRKY34	0.66	0.3269		0.2725	0.0004	down	0.371	0.0004	down	0.2933	0.0002	down	1.5186	0.0204	up	0.7985	0.4995	
BdWRKY35	1.1683	0.1463		1.9512	0.0009	up	1.9062	0.0024	up	1.1373	0.5073		1.6072	0.0236	up	0.7976	0.0111	
BdWRKY36	0.3123	3E-06	down	0.1425	4E-05	down	0.1306	3E-05	down	0.3151	2E-05	down	0.1737	5E-05	down	0.1588	0.0005	down
BdWRKY37	1.9913	0.0043	up	0.4725	0.0302	down	2.063	0.0107	up	1.0062	0.1032		2.3281	0.0318	up	4.2749	0.005	up
BdWRKY38	0.4636	1E-05	down	0.451	0.0001	down	0.741	4E-05		0.7898	0.1222		0.9675	0.0352		0.4658	1E-06	down
BdWRKY39	1.2477	0.2543		2.3117	0.0032	up	2.7271	0.0026	up	2.364	0.0022	up	2.4455	0.0036	up	2.2849	0.0026	up
BdWRKY40	0.4437	0.0137	down	1.6938	0.0246	up	2.4837	0.0033	up	0.5068	0.0157	down	2.1126	0.0084	up	0.9804	0.5867	
BdWRKY41	0.2012	1E-05	down	0.13	0.001	down	0.3652	0.0002	down	0.2587	1E-05	down	1.4594	0.7136		0.2157	3E-06	down
BdWRKY42	0.4945	0.0036	down	0.3284	0.0003	down	0.5898	0.0586		0.7886	0.0081		0.7231	0.2792		0.2385	0.0118	down
BdWRKY43	0.4854	0.0016	down	0.5618	0.0003	down	1.0549	0.1788		0.6521	0.0008	down	0.928	0.0273		0.4793	0.0001	down
BdWRKY44	0.595	0.0617		0.294	0.0035	down	0.5778	0.0477	down	0.6515	0.0812		0.733	0.3566		1.4539	0.2049	
BdWRKY45	0.6921	0.0608		1.3481	0.1635		2.6136	0.0128	up	1.3279	0.1499		1.5491	0.0274	up	1.5233	0.0463	up
BdWRKY46	0.4421	0.0797		0.8672	0.3143		2.6357	0.0029	up	2.2446	0.0103	up	4.0344	0.0199	up	1.8242	0.4953	
BdWRKY47	0.1636	0.0008	down	0.6065	0.1158		0.6988	0.0389		0.1998	0.0035	down	0.6646	0.0605		0.2721	0.0013	down
BdWRKY48	1.3058	0.0156		2.2323	7E-05	up	2.3606	8E-05	up	1.4503	0.0873		3.336	0.0002	up	2.9649	2E-05	up
BdWRKY49	0.2281	0.0004	down	0.2052	0.0003	down	0.5096	0.0034	down	0.0042	0.0003	down	0.0073	0.0002	down	0.0091	1E-06	down
BdWRKY50	0.3703	0.0001	down	0.4971	0.0001	down	1.617	0.0024	up	0.7105	0.0006		1.472	0.0853		0.5186	0.0001	down
BdWRKY51	0.2007	5E-07	down	0.1874	1E-07	down	0.6262	0.0002	down	0.2222	0.0002	down	0.4206	5E-06	down	0.2475	2E-07	down
BdWRKY52	0.3859	0.0004	down	0.5121	2E-05	down	1.8413	1E-04	up	0.57	0.0006	down	0.977	0.0341		0.7661	0.0116	
BdWRKY53	0.2396	0.0023	down	0.2134	0.0009	down	0.6547	0.0452	down	0.5772	0.0313	down	0.8579	0.1456		0.2286	0.0015	down
BdWRKY54	0.271	0.0004	down	0.273	0.0007	down	0.4505	0.0017	down	0.4686	0.0136	down	0.8005	0.3277		0.2644	0.0028	down
BdWRKY55	0.8062	0.8075		0.241	0.0027	down	0.4233	0.0138	down	0.5098	0.0163	down	0.748	0.336		0.1503	0.0009	down
BdWRKY56	0.2004	6E-05	down	0.2973	7E-06	down	0.8384	0.0012		0.2877	5E-06	down	0.4376	9E-05	down	0.2816	7E-05	down
BdWRKY57	0.8531	0.0035		1.2633	0.094		2.6027	0.0002	up	1.2651	0.0075		2.1626	4E-05	up	0.8872	0.032	
BdWRKY58	0.0644	5E-07	down	0.14	1E-06	down	0.2319	7E-06	down	0.1064	2E-06	down	0.129	5E-05	down	0.0607	0.0002	down

BdWRKY59	21.862	0.0023	up	0.2362	0.1148		1.0315	0.4876		1090.6	0.0002	up	2.3281	0.3904	up	0.9899	0.2819	
BdWRKY60	0.6512	0.0008	down	0.6007	0.0003	down	1.4537	0.0256		1.041	0.8854		1.4117	0.0496		0.6084	0.0003	down
BdWRKY61	0.5052	0.0007	down	0.3719	0.0001	down	1.2305	0.4893		0.9864	0.436		1.6326	0.012	up	0.4573	0.0003	down
BdWRKY62	0.2376	0.0028	down	0.2439	0.0003	down	0.3375	0.0011	down	0.2237	0.0028	down	0.3417	0.0013	down	0.294	0.0011	down
BdWRKY63	0.5708	0.0004	down	0.6223	0.0025	down	1.2267	0.6532		1.4363	0.0093		1.6809	0.0065	up	0.589	0.0006	down
BdWRKY64	0.4999	0.0013	down	0.4809	0.001	down	0.768	0.0142		0.6631	0.0073		0.7413	0.0061		0.4122	0.0005	down
BdWRKY65	0.9956	0.0585		495.12	2E-07	up	0.5157	0.1398		1.0062	0.1856		2.3281	0.0006	up	0.9899	0.4422	
BdWRKY66	0.3077	0.0002	down	0.1996	6E-06	down	0.3734	1E-05	down	0.4097	0.0054	down	0.6823	0.0014		0.3296	0.0012	down
BdWRKY67	0.2334	3E-05	down	0.2439	2E-05	down	0.468	0.0025	down	0.3871	6E-05	down	0.4824	0.0002	down	0.3283	2E-05	down
BdWRKY68	0.2645	0.007	down	0.6072	0.0019	down	1.0613	0.1135		0.2358	0.0053	down	0.6283	0.0025	down	0.3404	1E-05	down
BdWRKY69	1841.8	1E-07	up	0.426	0.0003	down	0.3132	0.0008	down	0.001	5E-07	down	0.2165	0.0013	down	0.0866	0.0004	down
BdWRKY70	1.5116	0.0313	up	0.3957	0.0032	down	0.6104	0.0541		0.9305	0.4859		0.3549	0.0015	down	0.4523	0.0277	down
BdWRKY71	0.3486	0.0027	down	0.0925	0.0007	down	0.2305	0.0001	down	0.1866	5E-05	down	0.312	0.0003	down	0.1255	2E-05	down
BdWRKY72	1.1998	0.0019		1.2357	0.005		2.516	9E-06	up	1.2628	0.0119		1.6032	0.0038	up	0.735	0.0035	
BdWRKY73	0.7206	0.0327		0.4469	8E-06	down	0.7178	0.0008		0.902	0.1252		1.0086	0.1008		0.5098	0.0045	down
BdWRKY74	0.5224	0.0002	down	0.3757	0.0003	down	0.6137	0.0004	down	0.8546	0.0307		0.8098	0.0059		0.5083	0.0015	down
BdWRKY75	0.0012	0.0002	down	0.0884	0.0026	down	0.0013	0.0003	down	0.0591	0.0903	down	0.0018	0.0001	down	0.0087	5E-05	down
BdWRKY76	1.505	0.0243	up	1.0965	0.8494		3.2495	0.0076	up	1.9031	0.0481	up	2.2638	0.0462	up	1.9118	0.0419	up
BdWRKY77	0.9067	0.3785		0.9719	0.3075		2.4416	0.0004	up	1.8354	0.0004	up	1.7721	0.0004	up	0.6361	0.0039	down
BdWRKY78	0.9225	0.7303		0.8216	0.5079		1.7694	0.0269	up	1.0325	0.9601		1.5119	0.0458	up	1.0103	0.9128	
BdWRKY79	0.2713	2E-10	down	0.297	5E-09	down	0.764	0.001		0.3976	3E-06	down	0.765	7E-05		0.3832	2E-05	down
BdWRKY80	1.8852	0.047	up	1.3413	0.3472		2.6243	0.0124	up	2.5175	0.0271	up	1.783	0.0372	up	2.1193	0.0254	up
BdWRKY81	784.23	3E-07	up	1120.6	2E-08	up	770.32	4E-09	up	544.57	4E-07	up	822.29	8E-09	up	555.2	5E-09	up
BdWRKY82	2.6598	4E-06	up	1.5374	0.0023	up	4.5596	4E-07	up	2.516	0.0048	up	3.2525	1E-05	up	1.7649	0.0004	up
BdWRKY83	0.7184	0.1445		0.6634	0.1003		1.1127	0.9027		0.8994	0.4973		1.0337	0.5551		1.1018	0.8486	
BdWRKY84	0.509	0.4538		0.4866	0.0144	down	1.3001	0.0001		0.0015	4E-07	down	0.0017	5E-07	down	0.9468	0.032	
BdWRKY85	0.4504	0.5957		0.4517	0.2631		0.3946	0.1427		0.8008	0.094		0.0237	1E-05	down	0.484	0.0025	down
BdWRKY86	0.132	0.0001	down	0.1759	4E-06	down	0.2981	0.0004	down	0.1654	4E-09	down	0.2075	8E-05	down	0.4643	0.0003	down

Supplementary table 4B. Expression data of the BdWRKY family genes after abiotic stress treatment.

gene name	Heat			Clod			NaCl			PEG			H_2O_2		
	fold-change	p-value	ragulation	fold-change	p-value	ragulation	fold-change	p-value	ragulation	fold-change	p-value	ragulation	fold-change	p-value	ragulation
BdWRKY1	2.0332	0.0003	up	0.6301	0.0075	down	0.3348	0.0001	down	0.0843	1E-05	down	0.0524	0.001	down
BdWRKY2	0.8185	0.0276		1.5569	0.0345	up	0.575	0.0004	down	0.4831	0.0025	down	0.3053	1E-05	down
BdWRKY3	1.0155	0.315		2.278	0.0047	up	0.7505	0.0067		0.4865	0.0014	down	0.7198	0.0146	
BdWRKY4	1.8394	9E-06	up	3.1034	0.0032	up	0.9006	0.2478		0.5833	1E-05	down	0.5951	3E-08	down
BdWRKY5	0.3969	0.0003	down	1.0074	0.5268		0.3474	0.0002	down	0.182	0.0002	down	0.0671	3E-05	down
BdWRKY6	2.2214	0.0003	up	4.6697	0.0002	up	0.9395	0.0025		0.4525	0.0016	down	0.5322	3E-06	down
BdWRKY7	0.1634	8E-05	down	0.6064	0.0053	down	0.1787	1E-05	down	0.1169	8E-07	down	0.0385	5E-06	down
BdWRKY8	1.9992	7E-07	up	3.023	7E-05	up	0.6601	9E-05		0.678	0.0308		0.5619	1E-05	down
BdWRKY9	1.2538	3E-08		1.1936	0.0844		0.5475	0.0003	down	0.2127	1E-04	down	0.0887	2E-06	down
BdWRKY10	6.1969	5E-05	up	6.5189	8E-06	up	2.1608	4E-07	up	1.1189	0.0733		2.3738	9E-05	up
BdWRKY11	1.2483	0.0253		2.3951	0.0002	up	0.379	6E-05	down	0.2932	9E-05	down	0.6012	0.0011	down
BdWRKY12	2.2585	0.018	up	3.16	0.0001	up	0.62	8E-05	down	0.3552	2E-05	down	0.3752	0.0002	down
BdWRKY13	1.5089	0.0193	up	3.309	0.0004	up	0.9445	0.2628		0.3592	0.0073	down	0.882	0.3633	
BdWRKY14	0.4528	0.0006	down	1.2469	0.2144		0.1846	6E-05	down	0.3661	0.0001	down	0.46	0.0002	down
BdWRKY15	4.0475	0.0004	up	2.6192	0.0003	up	3.6067	0.0003	up	0.6429	0.0564		1.0633	0.6709	
BdWRKY16	1.8376	0.0045	up	4.5349	0.0002	up	1.0722	0.6637		0.5148	0.0053	down	0.6125	0.0012	down
BdWRKY17	2.8224	4E-05	up	4.9836	0.0008	up	1.4511	0.0145		1.406	0.0358		0.9651	0.7304	
BdWRKY18	1.6369	0.0375	up	2.3963	0.0253	up	0.6868	0.1113		0.5949	0.1173		0.7096	0.1949	
BdWRKY19	0.0646	3E-05	down	0.7671	0.1099		0.0786	8E-06	down	0.0359	2E-06	down	0.0539	2E-06	down
BdWRKY20	0.4325	0.002	down	1.5633	0.0376	up	0.2031	8E-09	down	0.3455	5E-05	down	0.2212	1E-06	down
BdWRKY21	0.9737	0.2817		0.9606	0.4722		0.8939	0.0122		0.2908	7E-05	down	0.8054	0.0025	
BdWRKY22	0.7586	0.1852		0.7347	0.1485		0.5191	0.0029	down	0.071	8E-05	down	0.3549	0.0002	down
BdWRKY23	1.5045	0.0089	up	1.1262	0.5027		0.2903	0.0002	down	0.2107	2E-05	down	0.3091	9E-06	down
BdWRKY24	0.8016	0.0003		2.1525	0.0008	up	0.474	0.0005	down	0.4195	4E-05	down	0.6224	8E-10	down
BdWRKY25	92.043	3E-06	up	2.8772	0.0064	up	1.1423	0.2937		0.6861	0.5201		1.1374	0.2088	
BdWRKY26	0.4109	2E-05	down	0.7593	0.0018		0.6098	0.0001	down	0.4107	0.0015	down	0.7635	0.0405	

BdWRKY27	1.9238	4E-06	up	1.5175	0.036	up	0.7383	0.1751		0.1955	0.0072	down	0.3338	6E-06	down
BdWRKY28	1.5085	0.0152	up	0.9877	0.5193		0.5638	0.0068	down	0.51	0.0054	down	0.3315	0.0015	down
BdWRKY29	7.4704	0.0007	up	3.9061	0.0002	up	0.8049	0.0092		0.4223	0.0169	down	0.9758	0.3477	
BdWRKY30	0.9141	0.1325		1.8983	0.0003	up	0.7515	0.0002		0.4599	0.0002	down	0.5391	0.0005	down
BdWRKY31	0.2015	2E-05	down	1.5474	0.0164	up	0.2933	5E-07	down	0.1641	9E-06	down	0.3043	8E-07	down
BdWRKY32	0.882	0.0301		2.7351	0.0021	up	0.4032	4E-06	down	0.2231	0.0012	down	0.0279	0.0016	down
BdWRKY33	4.979	0.0024	up	4.873	0.0002	up	1.2632	0.1115		0.289	0.0006	down	0.7569	0.1546	
BdWRKY34	0.7293	0.5731		1.2652	0.6999		0.3684	0.1472	down	0.1557	0.0255	down	0.2013	0.0366	down
BdWRKY35	11.99	2E-05	up	1.5137	0.0103	up	0.4069	0.0014	down	0.3818	0.0159	down	0.8295	0.2502	
BdWRKY36	1.6352	0.0074	up	2.5382	0.0003	up	0.7332	0.0046		0.5565	0.0048	down	0.5281	0.0026	down
BdWRKY37	7.9046	0.0021	up	2.3244	0.0402	up	2.0532	0.0207	up	0.8045	0.2919		0.7173	0.1488	
BdWRKY38	0.9859	0.0242		2.975	4E-06	up	0.4975	3E-05	down	0.2978	0.0001	down	0.4558	3E-06	down
BdWRKY39	2.2962	0.0002	up	1.7041	0.0013	up	0.5523	0.0003	down	0.2839	1E-05	down	0.2487	0.0001	down
BdWRKY40	1.5026	0.0459	up	1.7819	0.0003	up	0.5283	4E-05	down	0.2193	1E-05	down	0.259	7E-08	down
BdWRKY41	1.7508	0.0042	up	3.4814	0.0003	up	0.3423	0.0011	down	0.205	0.0017	down	0.2935	0.0011	down
BdWRKY42	1.7169	0.0367	up	4.8904	0.002	up	0.9905	0.9196		0.3662	0.0298	down	0.539	0.0603	down
BdWRKY43	1.5157	0.0018	up	1.6199	0.0021	up	0.7257	0.083		0.5735	0.0103	down	0.8853	0.6317	
BdWRKY44	2.48	0.0046	up	3.1177	0.0002	up	0.8244	6E-06		0.4052	2E-05	down	0.4273	0.0009	down
BdWRKY45	1.5485	0.0254	up	2.0052	0.0146	up	0.7922	0.0992		0.3895	0.0055	down	0.316	0.0012	down
BdWRKY46	1.5027	0.0352	up	0.9188	0.0178		0.9096	0.0543		0.2823	0.0002	down	0.2509	5E-05	down
BdWRKY47	1.9172	0.0002	up	2.1604	3E-05	up	0.52	0.0007	down	0.4981	4E-05	down	0.3537	7E-06	down
BdWRKY48	2.1485	0.0004	up	3.098	0.0013	up	0.8473	0.0223		0.6707	0.0026		0.4008	4E-05	down
BdWRKY49	0.5966	0.0252	down	1.0239	0.343		0.8066	0.2966		0.4264	0.0002	down	0.2839	8E-05	down
BdWRKY50	7.462	3E-05	up	0.4396	0.0006	down	0.0893	4E-05	down	0.2189	0.0001	down	0.114	1E-05	down
BdWRKY51	0.1614	1E-05	down	0.2651	4E-05	down	0.2638	0.0003	down	0.0825	9E-07	down	0.1451	2E-06	down
BdWRKY52	0.13	3E-05	down	0.6014	0.0006	down	0.1052	2E-06	down	0.0824	5E-06	down	0.1772	5E-06	down
BdWRKY53	0.7061	0.0338		1.858	0.0058	up	0.9079	0.0101		0.3279	1E-06	down	0.5079	0.0002	down
BdWRKY54	0.566	0.0121	down	1.6828	0.0189	up	1.3363	0.1216		0.2481	0.0003	down	0.575	0.004	down
BdWRKY55	4.4023	0.0007	up	4.3448	6E-07	up	1.4172	3E-05		0.706	0.0026		0.5701	0.0001	down
BdWRKY56	2.7381	0.0004	up	2.2497	0.0002	up	0.5178	0.0003	down	0.3346	0.0003	down	0.7373	0.0019	
BdWRKY57	0.9771	0.3834		2.2919	0.0002	up	0.5295	0.0019	down	0.1677	1E-04	down	0.3527	0.0003	down
BdWRKY58	0.1483	8E-05	down	0.6552	0.0024	down	0.0745	9E-07	down	0.1098	9E-05	down	0.1418	3E-06	down

BdWRKY59	6.0223	0.0001	up	9.014	2E-05	up	2.6635	0.0002	up	0.5709	0.0047	down	0.8628	0.4593	
BdWRKY60	0.2652	7E-05	down	1.809	0.0023	up	0.4358	0.0004	down	0.2644	0.0003	down	0.2601	6E-05	down
BdWRKY61	1.1699	0.3967		2.7997	0.0001	up	0.5342	2E-05	down	0.3533	0.0002	down	0.436	7E-06	down
BdWRKY62	0.4797	1E-06	down	1.2973	0.039		0.073	1E-06	down	0.3188	0.0002	down	0.1077	5E-07	down
BdWRKY63	0.442	0.0006	down	3.0239	0.0162	up	0.6056	9E-06	down	0.3568	2E-05	down	0.277	4E-06	down
BdWRKY64	0.6951	5E-05		2.7119	3E-06	up	0.4146	0.0002	down	0.3168	0.0588		0.243	0.9424	
BdWRKY65	3.8172	0.0055	up	4.8832	0.0144	up	3.2325	0.0112	up	0.3737	0.045	down	1.2235	0.254	
BdWRKY66	4.3589	3E-05	up	2.7828	3E-05	up	0.5106	4E-05	down	0.2653	1E-05	down	0.3085	9E-06	down
BdWRKY67	0.9954	0.5417		3.1458	9E-05	up	0.7015	0.0148		0.4093	0.0006	down	0.3566	0.0002	down
BdWRKY68	3.6984	0.0409	up	3.5063	0.0152	up	0.2698	0.0228	down	1.83	0.013	up	0.7696	0.3868	
BdWRKY69	1.7412	0.0494	up	3.4877	0.0004	up	0.7119	0.0281		0.4371	0.0009	down	0.9674	0.4186	
BdWRKY70	0.3902	4E-06	down	0.6607	0.0107		0.5654	4E-05	down	0.1659	8E-05	down	0.1703	4E-06	down
BdWRKY71	1.0106	0.9962		0.8972	0.733		0.3993	0.0526		0.4008	0.0766	down	0.1931	0.0066	down
BdWRKY72	2.4994	0.0027	up	2.6878	0.0016	up	0.5526	0.0037	down	0.4182	0.0007	down	0.3765	0.0003	down
BdWRKY73	1.2749	0.0159		2.5083	0.0003	up	0.4421	0.0002	down	0.3534	5E-05	down	0.337	8E-05	down
BdWRKY74	0.653	0.0845		4.0877	6E-05	up	0.3509	0.0004	down	0.4882	0.004	down	0.1912	2E-05	down
BdWRKY75	2.6629	3E-05	up	3.2846	0.0002	up	1.1493	0.0379		0.2905	4E-06	down	0.1281	1E-05	down
BdWRKY76	0.6945	0.1493		1.4699	0.0363		0.8492	0.0355		0.361	0.0004	down	0.6932	0.0067	
BdWRKY77	2.3559	0.0337	up	3.2729	0.0269	up	1.5026	0.3429		0.7356	0.4345		1.3949	0.5858	
BdWRKY78	0.8144	0.0186		1.5643	0.0274	up	0.9827	0.0544		0.5842	0.0023	down	0.7116	6E-05	
BdWRKY79	1.5072	0.0016	up	3.032	0.0008	up	0.4383	5E-05	down	0.5568	0.0005	down	0.499	5E-05	down
BdWRKY80	3.2629	0.0001	up	4.7082	4E-05	up	1.5178	0.042	up	0.5678	0.0405	down	0.5799	0.0004	down
BdWRKY81	78.884	7E-06	up	1.0211	0.5803		11.989	8E-05	up	0.4325	0.0456	down	0.6717	0.0974	
BdWRKY82	1.1212	0.6462		1.8038	0.0009	up	0.8062	0.0072		0.2024	0.0007	down	0.3672	0.0162	down
BdWRKY83	0.2413	0.0036	down	0.5086	0.0002	down	0.2136	1E-06	down	0.1814	8E-05	down	0.2082	1E-05	down
BdWRKY84	4.4861	5E-05	up	1.2136	0.7112		3.0713	2E-05	up	0.2014	7E-07	down	2.002	1E-05	up
BdWRKY85	3.2396	0.0003	up	2.2692	6E-05	up	1.3535	0.0041		0.6051	0.0028	down	0.6443	0.003	down
BdWRKY86	0.6648	0.0016		0.3425	1E-05	down	0.1364	1E-06	down	0.1734	1E-05	down	0.2914	9E-05	down

Supplementary table 4C. Expression data of the BdWRKY family genes after phytohormone treatment.

gene	MeJA			6-BA			SA			ABA		
name	fold-change	p-value	regulation	fold-change	p-value	regulation	fold-change	p-value	regulation	fold-change	p-value	regulation
BdWRKY1	0.0604	0.0002	down	0.7272	0.0544		0.0597	3E-06	down	0.0684	3E-05	down
BdWRKY2	0.4506	0.0002	down	1.8288	0.0005	up	0.4392	5E-05	down	0.2545	0.0039	down
BdWRKY3	0.4661	0.0001	down	2.5057	0.0011	up	0.4315	9E-05	down	0.372	0.0016	down
BdWRKY4	0.4857	0.0004	down	1.5811	4E-05	up	0.3642	7E-08	down	0.1911	1E-05	down
BdWRKY5	0.1352	6E-05	down	0.6246	0.0044	down	0.1607	3E-05	down	0.0583	0.0002	down
BdWRKY6	0.3759	1E-04	down	1.2228	0.003		0.377	0.0099	down	0.2526	4E-06	down
BdWRKY7	0.1928	0.0001	down	0.1734	3E-06	down	0.1161	5E-07	down	0.0911	1E-05	down
BdWRKY8	0.2928	1E-07	down	2.182	0.0006	up	0.2852	0.0004	down	0.2762	1E-05	down
BdWRKY9	0.2215	0.0027	down	0.9885	0.488		0.108	1E-05	down	0.0604	0.0018	down
BdWRKY10	1.7735	0.016	up	4.3322	5E-06	up	1.5047	0.0063	up	0.1658	4E-06	down
BdWRKY11	0.4202	7E-05	down	1.671	0.0177	up	1.9632	0.0005	up	0.2409	1E-04	down
BdWRKY12	0.322	0.0034	down	1.5959	0.0134	up	0.2586	0.0005	down	0.2541	3E-07	down
BdWRKY13	0.687	0.0423		1.8903	5E-05	up	0.33	0.0003	down	0.1516	2E-05	down
BdWRKY14	0.4788	4E-05	down	1.4013	0.1512		13.935	7E-07	up	0.3931	0.001	down
BdWRKY15	1.8669	0.0458	up	2.2967	0.0009	up	0.9545	0.9868		0.1856	0.0002	down
BdWRKY16	0.3798	0.0001	down	2.1294	0.0021	up	0.3048	0.0036	down	0.2713	0.0011	down
BdWRKY17	0.5784	0.0003		2.2743	0.0192	up	0.4404	0.0026	down	0.1989	0.0006	down
BdWRKY18	0.2826	0.0075	down	1.7241	0.0372	up	0.2398	0.0016	down	0.4197	0.0018	down
BdWRKY19	0.1124	3E-06	down	0.158	5E-06	down	0.0767	8E-06	down	0.0229	5E-07	down
BdWRKY20	0.3853	2E-06	down	0.852	0.0173		0.3848	0.0008	down	0.0458	2E-07	down
BdWRKY21	0.202	1E-05	down	1.5965	0.0008	up	0.4063	0.0002	down	0.2442	0.0002	down
BdWRKY22	0.0853	0.0061	down	0.2813	0.0003	down	0.0617	7E-06	down	0.054	0.0002	down
BdWRKY23	0.1511	4E-05	down	0.6073	0.0044	down	0.1333	7E-05	down	0.1103	4E-05	down
BdWRKY24	0.32	6E-08	down	1.3997	0.029		0.2914	0.0003	down	0.2687	0.0012	down
BdWRKY25	2.3697	0.012	up	3.8433	0.0009	up	1.5127	0.0462	up	1.5387	0.02	up
BdWRKY26	0.5399	0.0002	down	1.0828	0.2816		0.462	2E-05	down	0.2251	0.0004	down
BdWRKY27	0.4546	2E-05	down	1.7738	0.0076	up	0.155	7E-05	down	0.094	2E-08	down

BdWRKY28	0.3433	0.0054	down	1.2984	0.318		0.5075	0.0055	down	0.0157	9E-06	down
BdWRKY29	0.2469	0.002	down	2.1831	0.0082	up	0.5134	0.0653		0.2967	0.0016	down
BdWRKY30	0.1751	4E-06	down	1.7528	3E-05	up	1.5529	0.0153	up	0.5021	0.0035	down
BdWRKY31	0.1929	3E-07	down	0.7383	0.0084		0.2116	3E-05	down	0.1651	3E-05	down
BdWRKY32	0.3021	3E-06	down	0.5003	4E-06	down	0.0552	0.0002	down	0.0678	0.0006	down
BdWRKY33	0.4803	0.003	down	1.2046	0.3805		0.3002	0.0002	down	0.4048	0.0283	down
BdWRKY34	0.1622	0.0276	down	0.5912	0.3172		0.118	0.0158	down	0.1071	0.015	down
BdWRKY35	0.7481	0.1231		0.8352	0.0755		0.1279	0.0002	down	0.4429	0.0005	down
BdWRKY36	0.5306	0.0007	down	1.9208	0.0028	up	0.2947	0.0006	down	0.2137	0.0002	down
BdWRKY37	0.6841	0.2163		1.6139	0.0325	up	0.2292	0.0023	down	0.1539	0.0017	down
BdWRKY38	0.2558	1E-06	down	1.5398	0.0046	up	0.3211	4E-06	down	0.2601	0.0005	down
BdWRKY39	0.2319	0.0004	down	1.5726	0.0103	up	0.3051	2E-05	down	0.2482	0.0007	down
BdWRKY40	0.3088	1E-05	down	1.014	0.4224		0.1743	3E-07	down	0.043	3E-06	down
BdWRKY41	0.2675	0.0003	down	1.7266	0.0335	up	0.2032	0.0002	down	0.1465	0.0012	down
BdWRKY42	0.1187	0.0045	down	3.4195	0.0098	up	0.9268	0.6529		0.5734	0.1124	
BdWRKY43	0.4615	0.0003	down	2.4369	0.0022	up	0.4841	0.0013	down	0.5344	0.0116	down
BdWRKY44	0.6242	0.0014	down	1.6765	0.0262	up	7.0313	1E-06	up	0.1237	0.0005	down
BdWRKY45	0.1097	0.0002	down	0.9531	0.7734		0.213	0.0016	down	0.1883	0.0003	down
BdWRKY46	0.1702	3E-06	down	0.8306	0.2331		0.1529	1E-05	down	0.1681	5E-05	down
BdWRKY47	0.2938	0.0002	down	1.051	0.9375		0.2924	3E-05	down	0.293	6E-05	down
BdWRKY48	0.3767	3E-05	down	0.923	0.8853		0.2792	4E-05	down	0.305	0.0006	down
BdWRKY49	0.2434	6E-05	down	1.2479	0.3618		0.3991	0.0005	down	0.9336	0.5848	
BdWRKY50	0.0275	4E-06	down	0.55	0.002	down	0.1114	2E-05	down	0.0808	0.0002	down
BdWRKY51	0.0357	6E-10	down	0.1538	6E-06	down	0.0264	3E-07	down	0.0279	7E-09	down
BdWRKY52	0.0377	1E-05	down	0.1708	0.0001	down	0.0753	1E-06	down	0.0228	1E-05	down
BdWRKY53	0.3119	0.0002	down	1.4078	0.0015		0.4562	0.0031	down	0.3078	9E-05	down
BdWRKY54	0.3082	0.0003	down	1.9195	0.0039	up	0.5636	0.0126	down	0.3668	0.0017	down
BdWRKY55	0.8073	0.0615		1.6712	0.0018	up	0.4844	0.0053	down	0.4022	0.0009	down
BdWRKY56	0.3159	8E-05	down	1.2996	0.0538		0.2271	0.001	down	0.2304	0.0014	down
BdWRKY57	0.2458	3E-05	down	0.8362	0.3999		0.2609	5E-05	down	0.3698	0.0061	down
BdWRKY58	0.292	6E-05	down	0.3632	0.0004	down	0.137	5E-06	down	0.3743	0.002	down
BdWRKY59	0.6572	0.084		4.0837	0.0009	up	0.4069	0.0031	down	0.0881	6E-06	down

BdWRKY60	0.2322	5E-05	down	0.9268	0.6634		0.3511	0.0002	down	0.3241	0.0013	down
BdWRKY61	0.2551	7E-06	down	0.96	0.0994		0.4181	1E-04	down	0.3381	0.0004	down
BdWRKY62	0.3471	1E-06	down	0.8125	0.0127		0.1727	2E-07	down	0.1771	3E-05	down
BdWRKY63	0.2493	9E-06	down	0.6041	2E-05	down	0.1668	2E-06	down	0.2289	3E-06	down
BdWRKY64	0.256	0.1294		0.7437	0.0001		0.1576	0.0081	down	0.264	0.2858	
BdWRKY65	0.4337	0.0703		2.0186	0.0315	up	0.6159	0.438		0.8101	0.4671	
BdWRKY66	0.1966	5E-06	down	0.7504	0.1142		0.206	2E-06	down	0.3012	0.0003	down
BdWRKY67	0.3321	0.0013	down	1.6991	0.0104	up	0.3713	0.0003	down	0.3545	0.0002	down
BdWRKY68	0.2422	0.0438	down	0.6587	0.4248		0.3044	0.0453	down	0.5192	0.226	
BdWRKY69	0.4842	0.0052	down	1.4113	0.013		0.3811	0.0011	down	0.2443	0.0013	down
BdWRKY70	0.266	5E-05	down	0.971	0.2459		0.1558	0.0001	down	0.0382	2E-07	down
BdWRKY71	0.1779	0.0134	down	0.3393	0.0715		0.0443	0.0008	down	0.3043	0.0257	down
BdWRKY72	0.362	0.0035	down	0.6782	0.035		0.1628	6E-05	down	0.1767	6E-05	down
BdWRKY73	0.5578	0.0005	down	0.8225	0.0983		0.1337	1E-05	down	0.1777	2E-05	down
BdWRKY74	0.1561	0.0001	down	1.0402	0.9378		0.2161	0.0007	down	0.3613	0.0015	down
BdWRKY75	0.396	8E-06	down	2.2778	0.0003	up	0.2005	0.0005	down	0.0978	1E-05	down
BdWRKY76	0.2004	0.0018	down	1.107	0.855		0.6585	0.0117	down	0.4313	0.0154	down
BdWRKY77	0.7285	0.4135		1.431	0.3853		0.3517	0.0373	down	0.4281	0.0697	
BdWRKY78	0.3241	4E-06	down	0.9835	0.0433		0.2731	0.0001	down	0.5431	0.0004	down
BdWRKY79	0.2923	6E-07	down	1.5577	0.0002	up	0.6958	0.0049		0.4154	1E-05	down
BdWRKY80	0.5697	0.0059	down	1.6135	0.0062	up	0.3518	0.0002	down	0.3253	0.0064	down
BdWRKY81	0.4806	0.0072	down	1.9188	0.0085	up	0.4842	0.0036	down	0.4049	0.002	down
BdWRKY82	0.2939	0.0011	down	0.7828	0.1776		0.2372	0.0019	down	0.091	0.0016	down
BdWRKY83	0.5066	0.0019	down	0.3664	0.0002	down	4.1154	1E-05	up	0.3274	0.0004	down
BdWRKY84	0.285	0.0007	down	0.47	5E-05	down	0.3605	1E-04	down	0.128	6E-07	down
BdWRKY85	0.6952	0.0026		2.1444	0.0019	up	0.5534	0.0009	down	0.261	2E-05	down
BdWRKY86	0.3968	6E-05	down	0.2535	4E-05	down	1.641	0.0043	up	0.1207	1E-05	down
