# Supplementary figures and images for: Genome-Wide Evolutionary Characterization and Expression Analyses of WRKY Family Genes in Brachypodium distachyon
Source: DNA Res. 2014 Jan 21;21(3):327–39. doi: 10.1093/dnares/dst060 (PMC4060952; doi:10.1093/dnares/dst060)

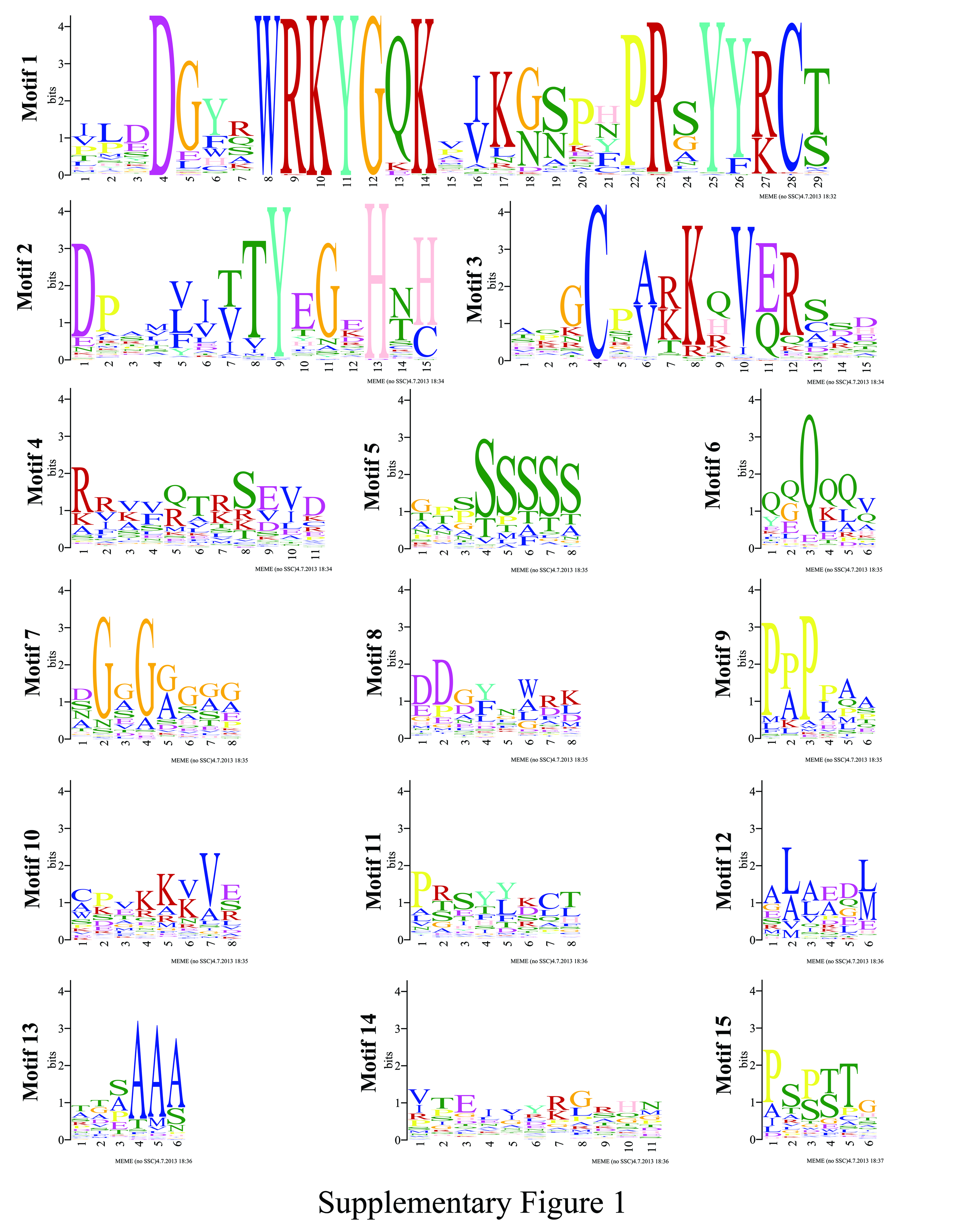

Supplement: Supplementary Data [file supp_dst060_dst060supp_fig1.tif]

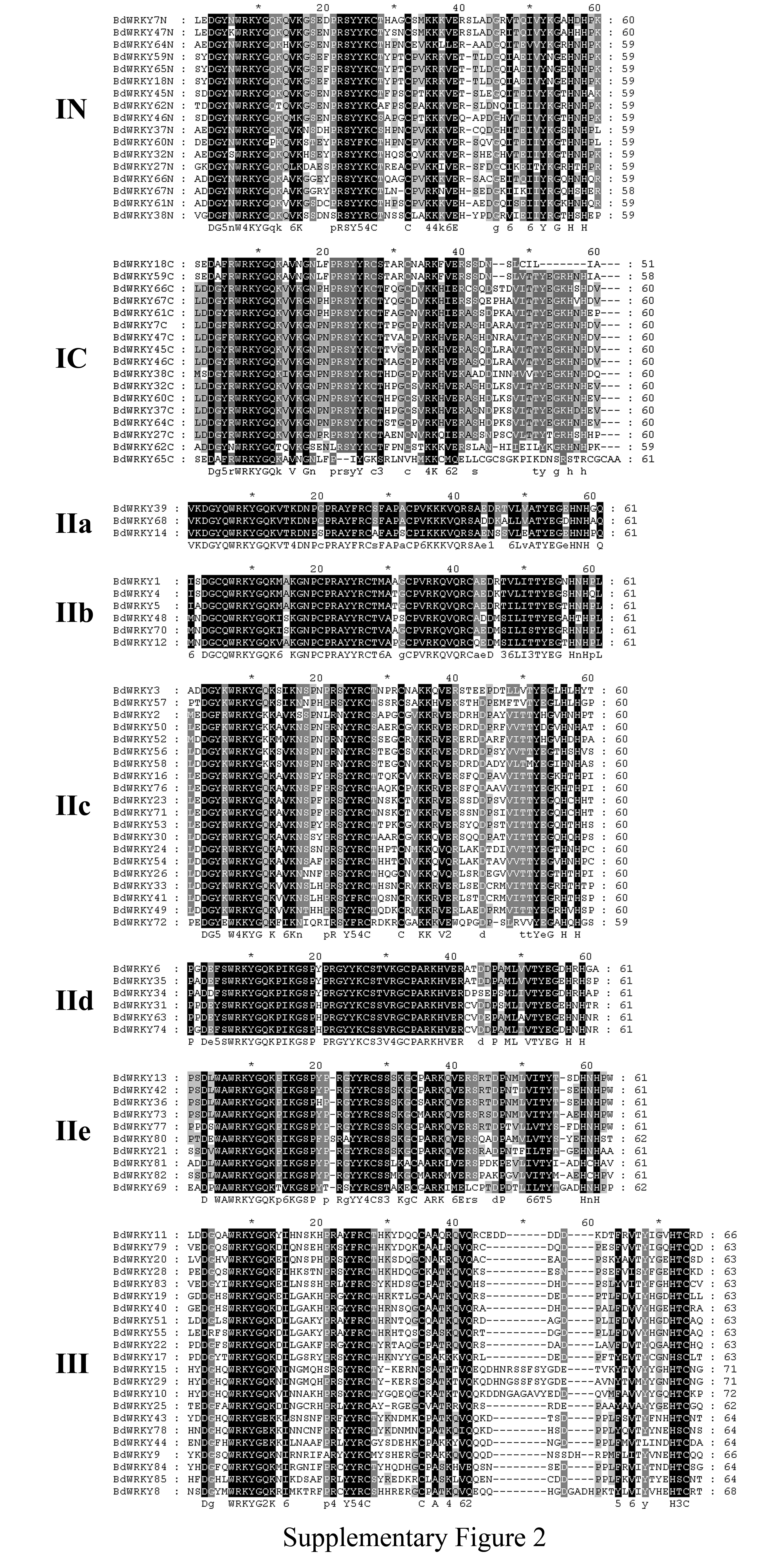

Supplement: Supplementary Data [file supp_dst060_dst060supp_fig2.tif]

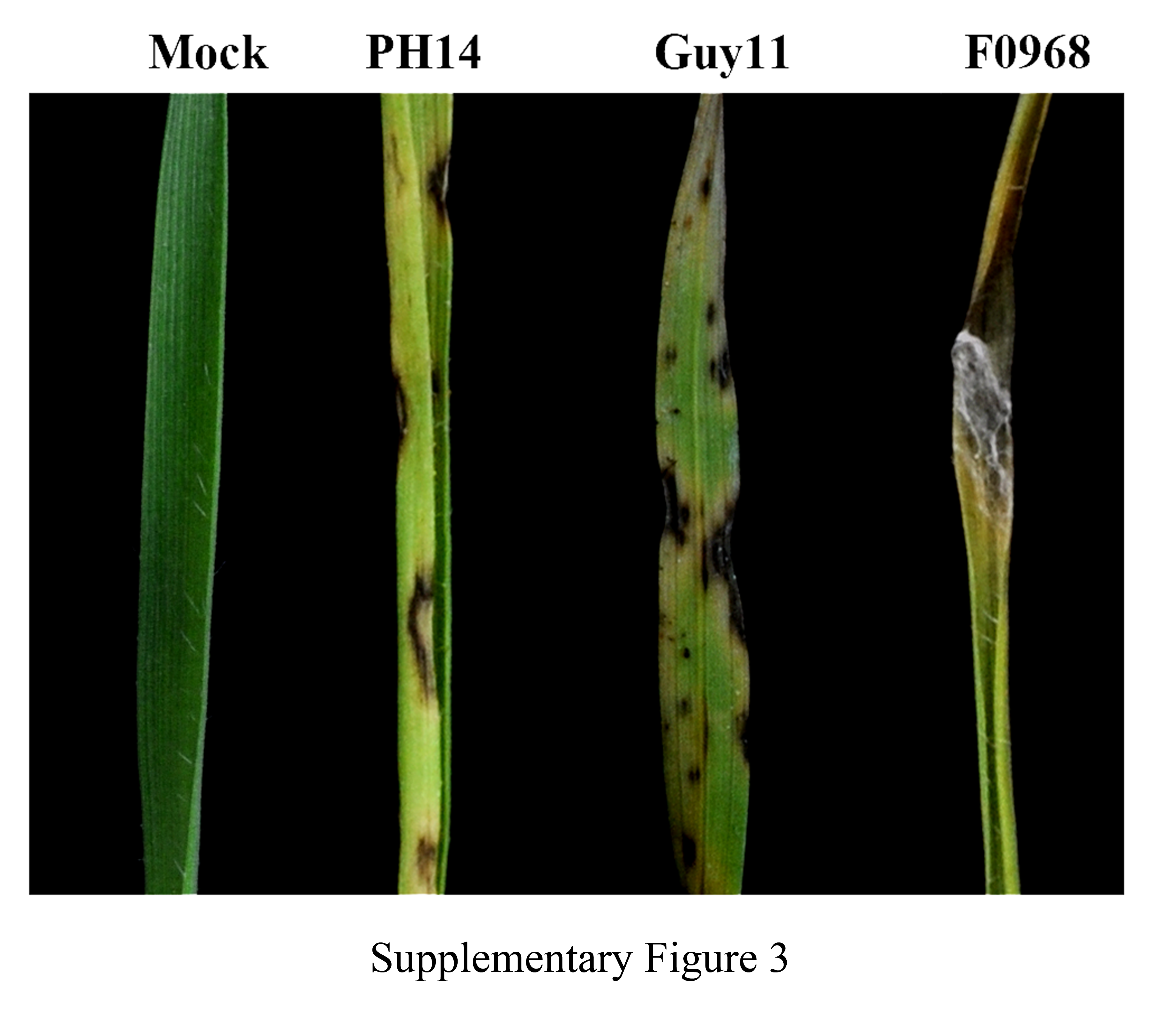

Supplement: Supplementary Data [file supp_dst060_dst060supp_fig3.tif]

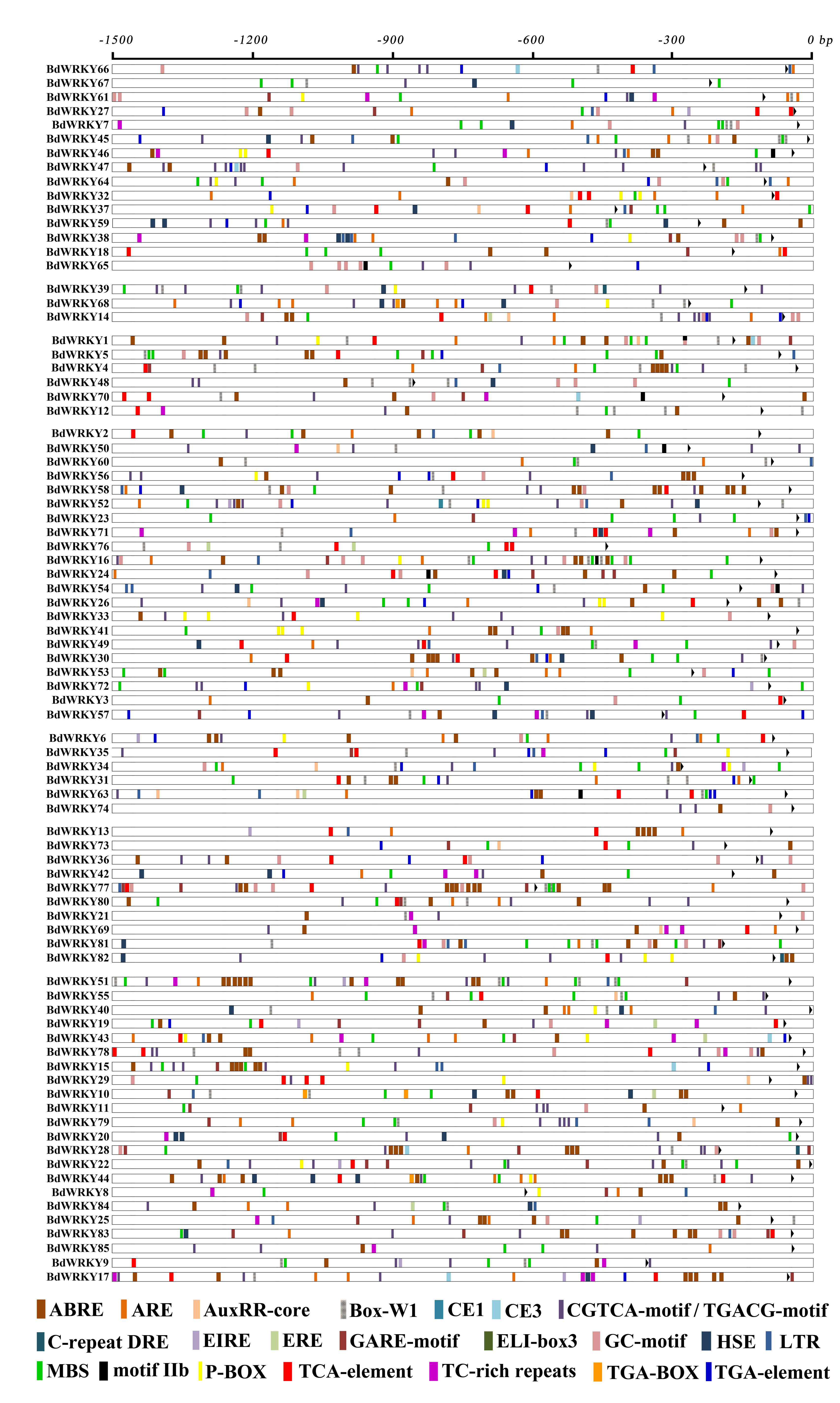

Supplement: Supplementary Data [file supp_dst060_dst060supp_fig4.tif]
